# Tailoring T_fh_ profiles enhances antibody persistence to a clade C HIV-1 vaccine in rhesus macaques

**DOI:** 10.7554/eLife.89395

**Published:** 2024-02-22

**Authors:** Anil Verma, Chase E Hawes, Sonny R Elizaldi, Justin C Smith, Dhivyaa Rajasundaram, Gabriel Kristian Pedersen, Xiaoying Shen, LaTonya D Williams, Georgia D Tomaras, Pamela A Kozlowski, Rama R Amara, Smita S Iyer

**Affiliations:** 1 Department of Pathology, School of Medicine, University of Pittsburgh Pittsburgh United States; 2 Graduate Group in Immunology, University of California, Davis Davis United States; 3 California National Primate Research Center, University of California, Davis Davis United States; 4 Department of Pathology, Microbiology, and Immunology, School of Veterinary Medicine, University of California, Davis Davis United States; 5 Department of Microbiology, Immunology, and Parasitology, Louisiana State University Health Sciences Center New Orleans United States; 6 Bioinformatics Core, Department of Pediatrics, UPMC Children's Hospital of Pittsburgh Pittsburgh United States; 7 Statens Serum Institute Copenhagen Denmark; 8 Center for Human Systems Immunology Durham United States; 9 Department of Surgery, Duke University Medical Center Durham United States; 10 Duke Human Vaccine Institute, Duke University Medical Center Durham United States; 11 Department of Molecular Genetics and Microbiology, Duke University Medical Center Durham United States; 12 Department of Integrative Immunobiology, Duke University Medical Center Durham United States; 13 Department of Microbiology and Immunology, Emory University Atlanta United States; 14 Yerkes National Primate Research Center, Emory University Atlanta United States; Osaka University Japan; The Feinstein Institute for Medical Research United States

**Keywords:** T-dependent B cell response, antibody persistence, vaccine efficacy, Rhesus macaque

## Abstract

CD4 T follicular helper cells (T_fh_) are essential for establishing serological memory and have distinct helper attributes that impact both the quantity and quality of the antibody response. Insights into T_fh_ subsets that promote antibody persistence and functional capacity can critically inform vaccine design. Based on the T_fh_ profiles evoked by the live attenuated measles virus vaccine, renowned for its ability to establish durable humoral immunity, we investigated the potential of a T_fh_1/17 recall response during the boost phase to enhance persistence of HIV-1 Envelope (Env) antibodies in rhesus macaques. Using a DNA-prime encoding gp160 antigen and T_fh_ polarizing cytokines (interferon protein-10 (IP-10) and interleukin-6 (IL-6)), followed by a gp140 protein boost formulated in a cationic liposome-based adjuvant (CAF01), we successfully generated germinal center (GC) T_fh_1/17 cells. In contrast, a similar DNA-prime (including IP-10) followed by gp140 formulated with monophosphoryl lipid A (MPLA) +QS-21 adjuvant predominantly induced GC T_fh_1 cells. While the generation of GC T_fh_1/17 cells with CAF01 and GC T_fh_1 cells with MPLA +QS-21 induced comparable peak Env antibodies, the latter group demonstrated significantly greater antibody concentrations at week 8 after final immunization which persisted up to 30 weeks (gp140 IgG ng/ml- MPLA; 5500; CAF01, 2155; p<0.05). Notably, interferon *γ*+Env-specific T_fh_ responses were consistently higher with gp140 in MPLA +QS-21 and positively correlated with Env antibody persistence. These findings suggest that vaccine platforms maximizing GC T_fh_1 induction promote persistent Env antibodies, important for protective immunity against HIV.

## Introduction

Despite options for testing and treatment, progress against HIV is slowing, emphasizing the urgent need for an HIV vaccine (Fauci, 2017; Eisinger and Fauci, 2018). While advances have been made in immunogen design and vaccine approaches, the main challenge lies in generating durable, high-affinity antibodies against the HIV-1 Envelope (Env) glycoprotein, crucial for protection. To design a vaccine that confers long-term protective immunity, effective stimulation of CD4 T follicular helper (T_fh_) cells is essential, as they provide vital costimulatory and cytokine support to B cells within germinal centers (GC), leading to the production of persistent antibodies following immunization (Slifka et al., 1998; Crotty, 2014). T_fh_ cells possess distinctive T_h_1, T_h_2, and T_h_17-type cell attributes, programmed by the inflammatory response during the T-cell priming phase, with each T_fh_ subset differentially contributing to GC B cell proliferation, survival, and differentiation (Morita et al., 2011; Barbet et al., 2018; Gao et al., 2023). Therefore, in addition to antigen selection, promoting the differentiation and expansion of optimal T_fh_ subsets to generate potent and enduring humoral immunity is critical for vaccine design.

Clinical studies have demonstrated that booster immunization with a T_h_1 glucopyranosyl lipid adjuvant-stable emulsion (GLA-SE)-formulated malaria antigen leads to enhanced antibodies at memory time points compared to an aluminum adjuvanted vaccine (Hill et al., 2019). We demonstrated that promoting the induction of T_fh_1 cells by utilizing interferon-induced protein (IP)10, a ligand for and an inducer of CXCR3, as a molecular adjuvant to a DNA vaccine (DNA_IP10_) followed by boosting with protein adjuvanted with Army Liposome Formulation (ALFQ) consisting of liposomal monophosphoryl lipid A (MPLA) plus a saponin derivative, QS-21 (Alving et al., 2012; Rao et al., 2018) enhanced GC responses, increased HIV anti-Env binding antibodies, and stimulated significantly higher cross-clade reactivity with increased specificity to V1V2 conformational epitopes, and higher avidity (Verma et al., 2019). Similarly, a recent macaque study utilizing Clade C DNA + GLA SE adjuvanted Env protein immunization revealed that frequencies of Env-specific CD28 + IFNγ+ cells, indicative of T_h_1 responses, correlated with the development of Env antibodies following the final immunization (Felber et al., 2020).

In addition to IFNγ, interleukin 17 (IL-17) has been shown to enhance cognate T-B cell interactions, leading to an effective GC response (Moyron-Quiroz et al., 2004; Mitsdoerffer et al., 2010; Kumar et al., 2013). Studies conducted in mice have further demonstrated that T_fh_17 cells support efficient antibody recall responses (Gao et al., 2023). Notably, the persistence of circulating T_fh_17 cells specific to measles has been observed in adults who have received the live attenuated measles virus vaccine (LAMV) during childhood, suggesting that measles vaccine triggers T_fh_17 responses which persist beyond the GC phase (Gao et al., 2023). Intriguingly, earlier studies exploring peripheral CD4 T cell determinants of serological memory elicited by LAMV have led to the hypothesis that LAMV promotes both T_h_1 and T_h_2 CD4 differentiation programs, based on production of IFNγ^+^ and IL-4, which are cytokines also produced by T_fh_ cells (Ovsyannikova et al., 2003a; Ward and Griffin, 1993; Ovsyannikova et al., 2003b). However, our understanding of the GC T_fh_ cell response to LAMV, which plays a crucial role in establishing long-term immunity against measles, remains incomplete.

In this study, based on GC T_fh_ profiles evoked by LAMV, we explored the potential of a T_fh_1 or mixed T_fh_1/17 targeted vaccine to enhance HIV-1 Env antibodies in rhesus macaques using an DNA-prime/protein boost approach. After booster immunization with a Clade C gp140 protein formulated in CAF01 (Wørzner et al., 2021), a cationic liposome-based formulation, we observed the generation of T_fh_1/17 cells within GCs, while T_fh_1 cells were induced when MPLA +QS-21 was used as the adjuvant. Notably, stimulating T_fh_1 cell induction with MPLA resulted in significantly greater antibody persistence at week 8 and up to 30 weeks after the final immunization. Moreover, the T_fh_1 regimen with MPLA adjuvant led to higher tier 1 neutralization titers, increased levels of IgG1 subclass antibodies, and improved antibody effector functions. These findings highlight a unique potential of T_fh_1 cells in promoting humoral immunity against HIV and suggest that vaccine strategies maximizing T_fh_1 cell induction may hold promise for eliciting durable protective immunity against HIV.

## Results

### GC T_fh_1 and GC T_fh_17 cells recalled by measles booster

**Figure 1.**
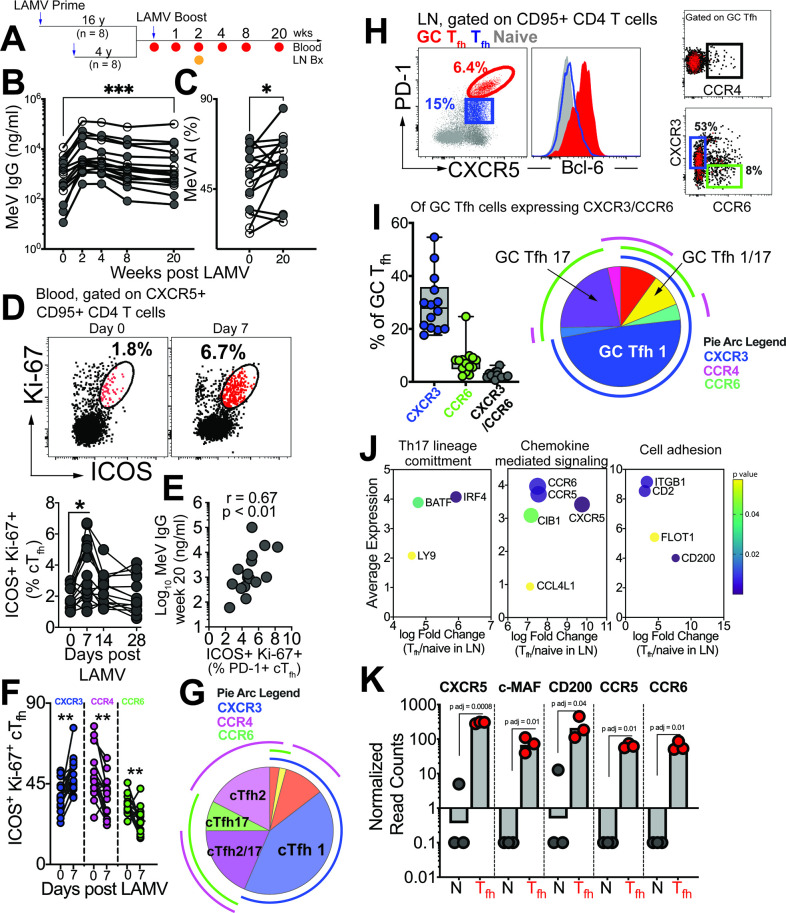
GC T_fh_1 and GC T_fh_17 cells recalled by measles booster. (**A**) Overview of study design; adult female rhesus macaques immunized with Live Attenuated Measles Virus vaccine (LAMV). (**B**) Serum MeV IgG kinetics measured by ELISA (filled circles, aged rhesus; open circles, young adults). (**C**) Avidity index (AI) of MeV IgG measured using chaotropic displacement ELISA with sodium thiocyanate at week 20 and week 0 in serum. (**D**) Representative flow cytometry plots showing ICOS^+^Ki-67^+^ circulating (c)Tfh cells in blood at day 0 and day 7 post LAMV. Kinetics of ICOS^+^Ki-67^+^ cTfh cells. (**E**) Correlation between ICOS + Ki-67+cTfh cells at day 7 and MeV IgG at week 20. (**F**) T_h_ profile of cTfh cells shows induction of CXCR3^+^ cTfh1 at day 7. (**G**) Boolean analysis (n=16) shows cTfh1 cells induced 1 week post LAMV. Overlapping pie arcs denote cTfh cells expressing multiple chemokine receptors as denoted by arc color. (**H**) Representative flow cytometry plots show CXCR5^+^ PD-1^++^ GC T_fh_ cells and histogram shows Bcl-6 expression on GC T_fh_ cells. T_h_ profile of GC T_fh_ cells shows expression of CXCR3 and CCR6. (**I**) Boolean analysis of GC T_fh_ cells expressing either CXCR3 or CCR6 (n=14) shows proportion of T_h_1, T_h_17 and T_h_1/17 GC T_fh_ subsets. (**J**) Bubble plots show genes for significantly enriched pathways related to T helper differentiation on sorted CXCR5 + PD-1+/++ cells. (**K**) Gene expression on sorted CXCR5 +PD-1+/++ cells. Statistical analysis was performed using two-tailed Wilcoxon matched-pairs signed rank test (in panels B-D, F) or spearman rank correlation test (**E**); * p<0.05, **p<0.01, *** p<0.001, **** p<0.0001.

To decipher the specific T_h_ subset of T_fh_ cells contributing to the establishment of durable serological memory, we employed live attenuated measles virus vaccine (LAMV) as a model and investigated T_fh_ responses in peripheral blood and lymph nodes (LN) in a cohort of 16 healthy adult female rhesus macaques (Figure 1A). Consistent with documented evidence of long-term persistence of the memory B cell response to LAMV, rapid recall of measles virus (MeV) IgG ensued at week 2 post LAMV, irrespective of the booster interval (Figure 1B). By week 20, MeV IgG concentrations displayed a 2.6-fold increase compared to baseline, signifying the efficacy of LAMV booster in generating persistent antibodies. These augmented levels of memory antibodies were accompanied by enhanced antibody avidity (Figure 1C), underscoring robustness of the humoral immune response elicited by LAMV.

**Figure 1—figure supplement 1.**
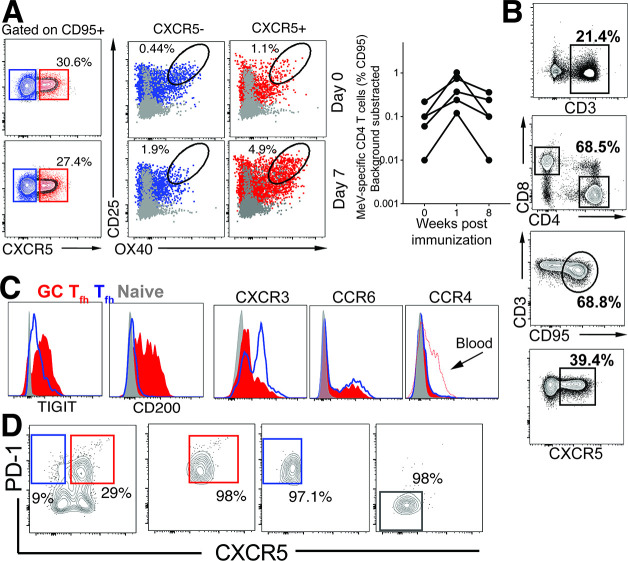
MeV specific Tfh cells. (**A**) Gating strategy to identify CXCR5^+^ OX40^+^ CD25^+^ MeV-specific T_fh_ cells within PBMCs following stimulation with MeV antigen at Day 0 and Day 7 post LAMV. Right panel illustrates the MeV specific responses in CD4 T cells (%CD95) at weeks 0, 1, and 8 following immunization. (**B**) Flow plots illustrate gating strategy to identify cTfh cells. (**C**) Representative histograms show expression of TIGIT, CD200, CXCR3, CCR6, and CCR4 on GC Tfh cells in LN. (**D**) Sorting schematic to isolate CXCR5 + PD-1+/++ cells for RNA sequencing.

Tracking MeV-specific T_fh_ cells using the activation-induced marker (AIM) assay showed induction of circulating (c)T_fh_ cells at day 7 post-vaccination (Figure 1—figure supplement 1A). Confirming T_fh_ cell induction in response to LAMV, activated cTfh cells, identified by co-expression of ICOS and Ki-67, were transiently upregulated at day 7 (Figure 1D, Figure 1—figure supplement 1B). These responding cTfh cells were PD-1 +and their frequencies at day 7, but not day 0, significantly correlated with MeV IgG at Week 20 (Figure 1E). We next proceeded to analyze heterogeneity within cTfh cells with respect to expression of T_h_1/2/17 chemokine receptors. Our temporal analysis of the responding cTfh compartment demonstrated significant induction of CXCR3 +T_fh_1 cells at day 7 (Figure 1F). In contrast, we observed a decrease in frequencies of CCR4-expressing cTfh2 subsets and CCR6-expressing cTfh17 subsets. Utilizing Boolean analysis, we further identified that the majority of responding cTfh cells expressed CXCR3, indicating induction of cTfh1 cells in response to LAMV (Figure 1G).

Expanding our investigation to include phenotypic and molecular features of CXCR5^+^ PD-1^++^ GC T_fh_ cells (Figure 1H, Figure 1—figure supplement 1C), we found that of the GC T_fh_ cells expressing either CXCR3 or CCR6, CXCR3 expression predominated (median %CXCR3+, 28%). Additionally, we observed that on average 7% of GC T_fh_ cells expressed CCR6. Notably, our analysis did not reveal substantial expression of CCR4 within GC T_fh_ cells (Figure 1I). Molecular analysis of sorted CXCR5 +PD-1+/++LN T_fh_ cells (Figure 1—figure supplement 1D) further provided insights into their functional specialization, with the induction of key transcription factors BATF and IRF4, as well as the expression of chemokine receptors CCR5 and CCR6, indicating the activation of T_h_1 and T_h_17 programs within GC T_fh_ cells (Figure 1J–K). Collectively, these findings demonstrate that both blood and GC T_fh_ cells, during the peak effector response following LAMV immunization, exhibit a predominant T_fh_1 helper bias, with GC T_fh_ cells also demonstrating a T_fh_17 bias.

### HIV-1 vaccine modalities for inducing GC T_fh_1 and GC T_fh_17 subsets

Building on our observations made with LAMV, we next investigated the potential of fine-tuning T_fh_ responses towards T_fh_1 and T_fh_17 profiles for generation of persistent anti-HIV-1 Env antibodies. We immunized two cohorts of rhesus macaques against HIV Env using a DNA prime and protein boost vaccination strategy tailored towards either a T_fh_1 (n=6) or mixed T_fh_1/17 (n=6) response (Figure 2—figure supplement 2). For both vaccine regimens, animals received an initial intradermal DNA prime vaccine encoding HIV-1 Env gp160 (C.1086C) at weeks 0, 4, 8, followed by an subcutaneous HIV C.ZA 1197 MB Env gp140 protein boost vaccination at weeks 12 and 20. To tailor vaccine induced T_fh_ responses, we included additional T_fh_ polarizing factors with DNA prime vaccines encoding for cytokines IP-10 (T_fh_1 regimen) or IP-10 and IL-6 (mixed T_fh_1/17 regimen), coupled with corresponding gp140 protein boost formulated in either the T_h_1 polarizing adjuvants, MPLA +QS-21, or the T_h_1/17 polarizing CAF01 adjuvant platform, respectively. Over the course of both the priming and boost stages, we performed routine collection of both blood and rectal secretions from animals to assess the extent of systemic and mucosal humoral immunity elicited by the two regimens. Additionally, we collected inguinal LN biopsies and fine needle aspirates of LN (FNA) at baseline, during the DNA prime stage, and following both protein boost to comprehensively characterize vaccine-elicited GC T_fh_ responses in draining LNs. GC responses typically peak between 2 and 3 weeks post first booster with more rapid recall kinetics after the second booster (Iyer et al., 2015). Our rationale for sampling LN 3 weeks following the second booster was to assess whether the reported effects of CAF01 on sustained antigen release might result in GC persistence (Henriksen-Lacey et al., 2011; Pedersen et al., 2020).

### Induction of robust T cell activation in GCs with HIV-1 Env formulated in CAF01 and MPLA+QS-21 adjuvants

**Figure 2.**
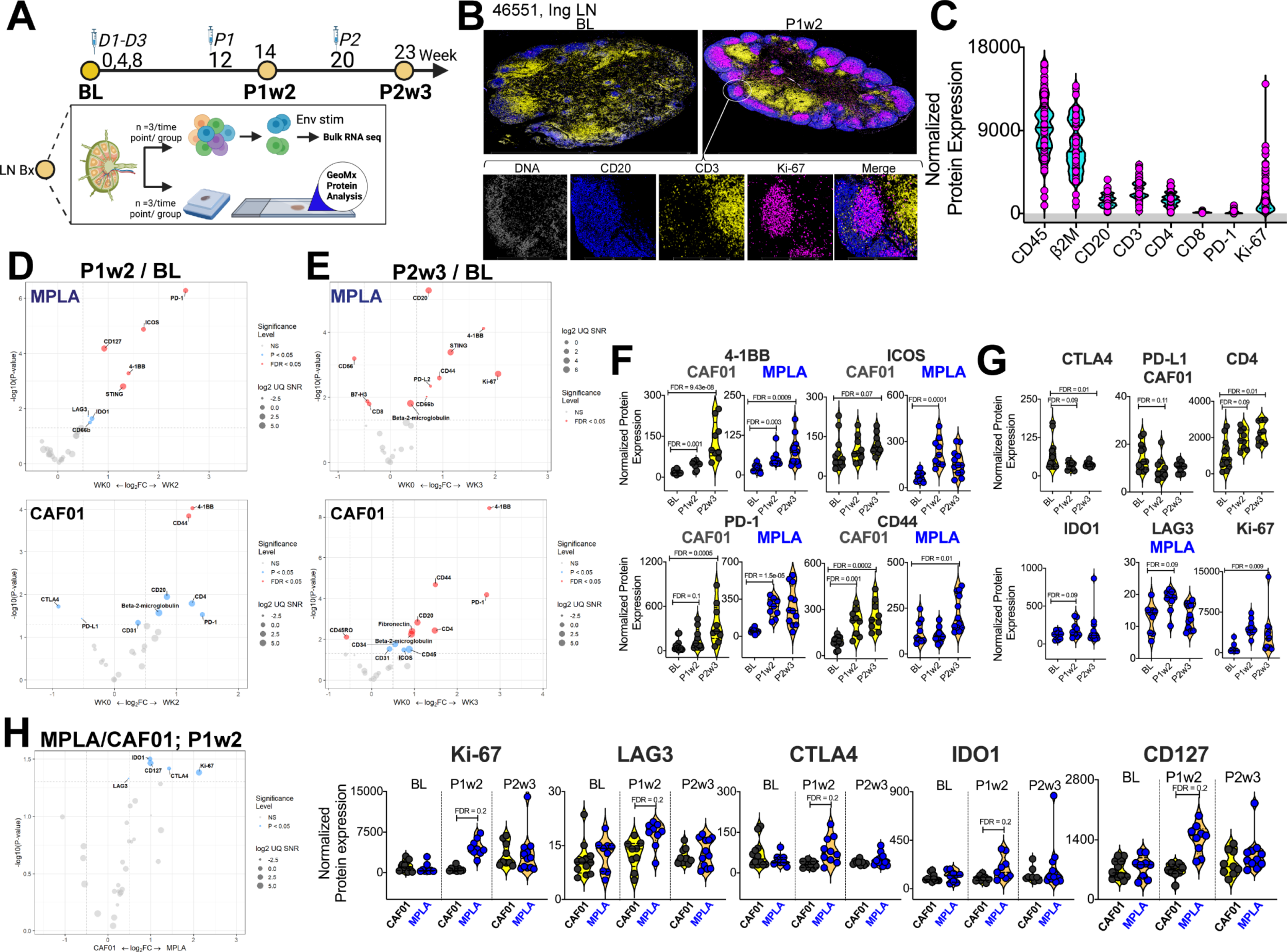
Induction of Robust T Cell Activation in GCs with HIV-1 Env Formulated in CAF01 and MPLA +QS-21 Adjuvants. (**A**) Overview of experimental design for in-situ proteomics and transcriptional analysis. (**B**) Representative images of GC from FFPE sections of inguinal LN at baseline and week 2 post protein 1 immunization with HIV-Env gp140 | MPLA +QS-21; scale bar; top, 3 mm, close up, 300 μm. LN sections were stained for CD20 (blue), CD3 (yellow), Ki-67 (magenta), and DNA (gray) to identify GCs. Circular ROIs (100 μm in diameter, total 60) were selected based on co-localization of CD3 with CD20 +Ki-67+GC B cells for proteomic profiling with a 32-plex antibody cocktail. Normalized protein expression (NPE) values were calculated using three negative control IgG probes. (**C**) NPE of key lineage markers across all ROIs at BL, P1w2, and P2w3. (**D–E**) Volcano plots show proteins induced post boost in each vaccine group. (**F**) Violin plots show common proteins induced post protein boost in MPLA and CAF01 groups. (**G**) Violin plots of proteins induced with CAF01 and MPLA. (**H**) Volcano plot (left) and Violin plots (right) of proteins significantly different across HIV-1 Env gp140 MPLA +QS-21 and CAF01 regimens. Differential expression was modeled using a linear mixed-effect model to account for the sampling of multiple ROI/AOI segments per patient/tissue. Criterion of significance was nominal p-value <0.05, plots show False discovery rate (FDR).

To gain insights into LN T_fh_ responses induced by MPLA and CAF01 platforms, we utilized complementary approaches of in-situ protein expression analysis of GCs and RNA sequencing of Env-stimulated CD95 +CD4 T cells of selected animals in each vaccine group (Figure 2A). To track immunological changes within GCs, we applied the GeoMx Digital Spatial Profiler to assess protein expression of key immune targets longitudinally at baseline (BL, prior to DNA immunization), week 2 post-protein 1 (P1w2) and week 3 post-protein 2 (P2w3) immunizations. We stained tissue sections with morphological markers (CD20, CD3, Ki-67) and collected circular regions of interest (ROI) based on co-localization of CD3 with CD20 +Ki-67+GC B cells. For each tissue, two to three ROIs were selected to ensure adequate representation of GCs within each animal. This approach allowed us to accurately identify GCs and analyze protein expression of both T and B cells within these specific regions (Figure 2B).

**Figure 2—figure supplement 1.**
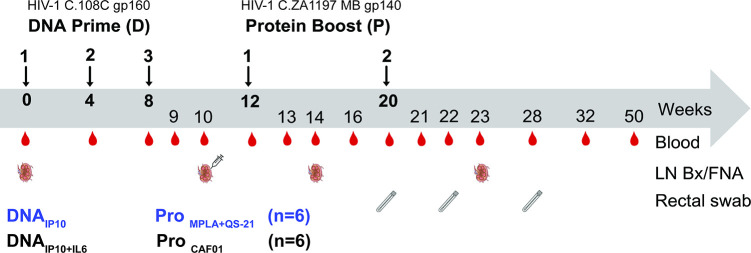
Experimental design of DNA-prime/Protein-boost Clade C HIV-1 immunization.

**Figure 2—figure supplement 2.**
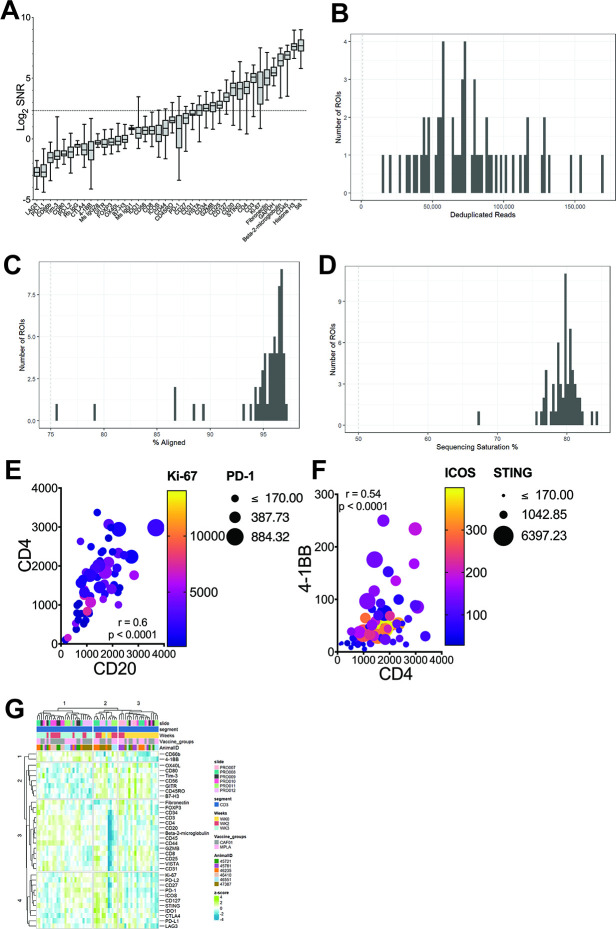
Sequencing QC histograms show (**A**) Signal-to-noise ratio computed by dividing raw count values by the geometric mean of the negative IgG probes. Quality control metrics- (**B**) deduplicated reads, (**C**) percent aligned reads, (**D**) sequencing saturation. (**E**) Bubble plot shows correlation of CD20 protein expression with CD4; bubble size corresponds to PD-1 expression, color intensity, Ki-67 expression; Spearman value, two-tailed p value of 60 pairs. (**F**) Bubble plot shows correlation of CD4 protein expression with 4-1BB; bubble size corresponds to ICOS expression, color intensity, STING expression; Spearman value, two-tailed p value of 60 pairs. (**G**) Heatmap plots show unsupervised hierarchical clustering of z-score values for each protein across time for each animal.

Thirty-two protein targets were profiled simultaneously, along with isotype controls (Ms IgG1, Ms IgG2A, Rb IgG) and housekeeping proteins (S6, Histone H3, GAPDH). After applying quality control measures, we calculated the signal-to-noise ratio for each target which provided robust assessment of protein expression levels (). CD45 and B2M were among the most abundantly expressed proteins while LAG3 and PD-L1 demonstrated low signal expression. To address variations in ROI surface area and tissue quality, normalized protein expression (NPE) was calculated for each protein. NPE analysis revealed robust expression of key lineage proteins, CD45, CD20, CD3, CD4, and CD8, as well as GC markers PD-1 and Ki-67 within ROIs (Figure 2C). We observed notable associations between protein expression patterns within the GC microenvironment; specifically, a strong correlation between the expression of CD4 and CD20 was observed (). Furthermore, a significant association between the co-stimulatory molecule 4-1BB and CD4 expression () was observed within the GC. Association of the innate immune protein stimulator of interferon genes (STING) with both 4-1BB and CD4 expression suggested a potential interplay between innate and adaptive immune pathways within the context of GC responses. Altogether, the protein expression data provide evidence for coordinated expression of key proteins involved in GC function in both vaccine groups ().

To identify specific proteins and pathways activated in response to vaccination, we performed differential expression analysis on a per-protein basis. We modeled NPE using a linear mixed-effect model (LMM), which allowed us to account for sampling multiple ROIs per tissue within each animal (FDR p value cut-off,<0.05). Volcano plots illustrate proteins induced at P1w2 (Figure 2D) and P2w3 (Figure 2E) following vaccination. Induction of 4-1BB, ICOS, PD-1, CD44 post boost indicated activation of T cells to antigen stimulation within GCs in both vaccine groups (Figure 2F). The higher relative expression of these proteins at P2w3 was consistent with the reported effects of CAF01 in GC persistence. We found that expression of the checkpoint inhibitors CTLA4 and PD-L1 was significantly reduced post CAF01 vaccination, while CD4 expression increased. In contrast, expression of the regulatory enzyme, IDO1; checkpoint inhibitor, PD-L2; and CD256 (B7-H3), and the innate sensor, STING, was significantly higher post vaccination in the MPLA group (Figure 2D–E and G).

Across vaccine regimens, significant differences were observed at P1w2 (Figure 2H). Significantly higher expression of Ki-67 with MPLA compared to CAF01 was suggestive of a more rapid GC response (Figure 2H). Furthermore, the checkpoint inhibitory receptors LAG3, CTLA4, as well as IDO1, exhibited higher expression levels with MPLA at P1w2. Importantly, no significant differences were observed at BL, validating the specificity of immune activation to vaccination. Altogether, the in situ protein analysis indicated robust induction of GC responses across vaccine platforms with distinct qualitative and quantitative effects initiated by MPLA versus CAF01 suggesting potential differences in regulatory mechanisms and immune activation pathways between the vaccine regimens.

### T_h_1 molecular programs potentiated by HIV-1 Env/MPLA+QS-21

**Figure 3.**
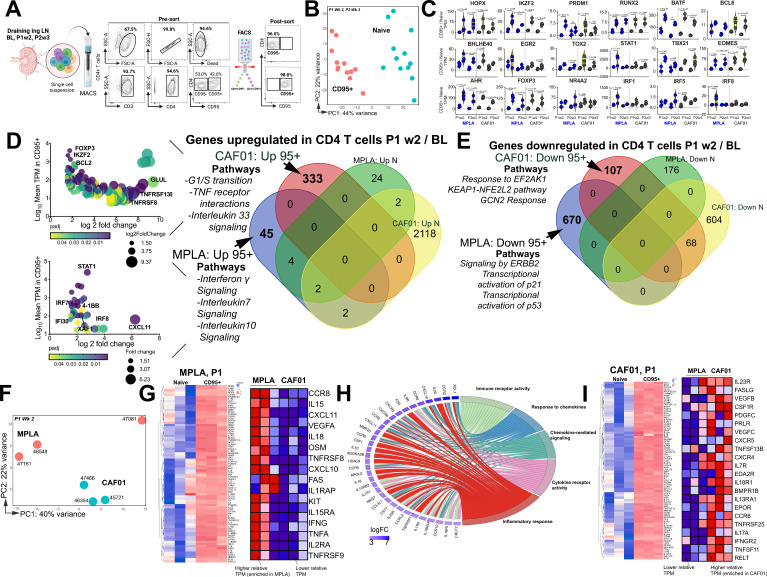
T_h_1 molecular programs potentiated by HIV-1 Env/MPLA + QS-21. (**A**) FACS purified CD95- (naive [N]) and CD95 + CD4 T cells, stimulated overnight with HIV-1 Env, were sequenced to assess transcriptional programs. (**B**) Principal component analysis (PCA) of all expressed genes shows distinct clustering of N and CD95 + cells. (**C**) Violin plots show transcripts per million (TPM) values of differentially expressed transcription factors (p adj <0.05) in CD95 + CD4 T cells relative to naive CD4 T cells post vaccination. (**D**) Bubble plots depict DEG at P1w2 versus BL in CD95 + CD4 T cells in CAF01 (top) and MPLA (bottom). Venn Diagram of DEG genes upregulated in CD95 + and naive subsets at P1w2 relative to BL shows 45 and 333 genes exclusively upregulated in CD95 + CD4 T cells with MPLA and CAF01, respectively. (**E**) Venn diagram of significantly downregulated genes. (**F**) PCA of all expressed genes shows distinct clustering across vaccine groups at P1w2. (**G**) Heat maps depict log_2_ gene expression (transcripts per million (TPM)) for highly DEG in CD95 + cells compared to naive cells at P1w2 in MPLA; heat map in inset depicts TPM of genes represented in Cytokine-cytokine receptor interaction pathways across vaccine regimens. (**H**) Chord plot shows GO Terms enriched with corresponding upregulated genes in CD95 + CD4 T cells at P1w2 with MPLA. (**I**) Heat maps depict log_2_ gene expression (TPM) for highly DEG in CD95 + cells compared to naive cells at P1w2 in CAF01; heat map in inset depicts TPM of genes represented in Cytokine-cytokine receptor interaction pathway across vaccine regimens.

To assess the extent of T_h_1/T_h_17 programming in CD4 T cells induced by vaccination, we profiled the transcriptome of CD95 +CD4 T cells isolated from the LN. We sort purified and RNA sequenced naive (CD95-) and CD95 + CD4 T cells at BL, P1w2, and P2w3 following overnight stimulation with HIV-1 Clade C Env peptide pools. A total of 18 samples per group, which included three biological replicates per subset, were sequenced in parallel resulting in over 25 million high-quality reads per sample (Figure 3A, Figure 3—figure supplement 1A-B). The transcriptomes of naive versus CD95 + CD4 T cells post immunization, in alignment with their distinctive biological states, exhibited clear demarcations along distinct dimensions within the principal component analysis (PCA) plot (Figure 3B). Transcription factors (TF) involved in regulating distinct cellular differentiation programs were enriched in CD95 + CD4 T cells at P1w2 and P2w3. These included HOPX, IKZF2, PRDM1, RUNX2, associated with effector differentiation. Additionally, TF such as BATF, BCL6, BHLHE40, EGR2, and TOX2 involved in T_fh_ differentiation were also identified. Furthermore, we identified genes regulating T_h_1 (STAT1, TBX21, EOMES), T_h_17 (AHR), and Treg (FOXP3, NR4A2) programs. Moreover, the expression of interferon regulatory factors (IRFs) controlling T cell differentiation (IRF1, IRF5, IRF8) was observed, demonstrating that CD95 + cells post vaccination encompassed effector and fully differentiated CD4 T cell subsets (Figure 3C).

**Figure 3—figure supplement 1.**
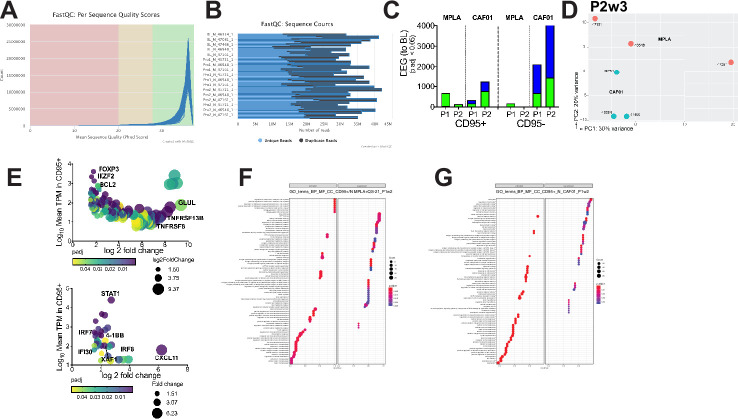
QC Metrics of RNA sequencing. (**A–B**) QC metrics (**C**) Bar graph shows DEG genes within CD4 subsets post vaccination relative to baseline. (**D**) PCA plot of CD95 +CD4 T cell subsets at P2w3 (**E**) Bubble plots show DEG enriched in CD95 +subsets at P1w2 in CAF01 and MPLA. GO-Terms of biological pathways differential induced in MPLA (**F**) and CAF01 (**G**) in CD95 +CD4 T cells at P1w2.

We first assessed temporal changes in gene expression by focusing on differentially expressed genes (DEGs) within CD95 + cells at P1w2 and P2w3, compared to BL (Venn diagram in 3D). Notably, a significantly higher level of transcriptional perturbation was observed in response to CAF01, particularly at P2w3, within both naive and CD95 + CD4 T cells (top bubble plot in 3D, Figure 3—figure supplement 1C), suggesting that CAF01 induces extensive gene expression changes in a cell-extrinsic manner. By specifically examining genes induced exclusively in CD95 + cells, but not naive cells, at P1w2, we made several observations. The T_h_1 transcriptional regulator STAT1, as well as IFNγ-induced genes such as IRF7, IRF8, IFI30, XAF-1, and CXCL11, were among the most highly expressed genes in MPLA (bottom bubble plot, 3D), highlighting the activation of T_h_1 programs following vaccination. Indeed, interrogation of top 3 enriched pathways utilizing the Reactome database (Fabregat et al., 2017) revealed that genes encompassing IFNγ, IL-7, and IL-10 signaling networks were induced with MPLA at P1w2. In the CAF01 group, we observed enrichment of co-stimulatory molecules TNFRSF8 (CD30) and TNFRS13B (TACI), expressed by activated T cells, along with expression of the anti-apoptotic molecule BCL-2 and the transcription factor Foxp3.

Analysis of genes downregulated showed pathways regulating cell cycle inhibitors p21 and p53 were enriched with MPLA while pathways regulating cellular responses to stimuli and stress were downregulated with CAF01 (Figure 3E). Collectively, transcriptional profiles demonstrated activation of effector programs within CD4 T cells with common and unique pathways induced across vaccine platforms.

Consistent with this, CD95^+^ CD4 T cells at P1w2 and P2w3 exhibited notable clustering patterns across vaccine regimens. Particularly, tighter, and more distinct clusters were observed at P1w2 compared to P2w3 notably with CAF01 (Figure 3F, Figure 3—figure supplement 1D-E). Exploration of genes regulating T_h_1 and T_h_17 programs within CD95 + CD4 T cells at P1w2 showed that CD95 + CD4 T cells in both MPLA and CAF01 groups exhibited overlapping expression profiles, with genes regulating major classes of cytokine-cytokine receptor interactions represented. The MPLA group exhibited higher expression levels of IFN-induced chemokines CXCL10 and CXCL11, IL1 family members IL18 and IL1RAP, IL15 family members IL15 and IL15RA, as well as TNF cytokine family members FAS, TNFα, TNFRSF8 (CD30), and TNFRSF9 (4-1BB) (Figure 3G–H, Figure 3—figure supplement 1F).

In contrast, the CAF01 group demonstrated expression of the T_h_17 receptor IL23R, along with TNF family members FASLG, TNFRSF13B (TACI), TNFRSF11 (RANKL), and TNFRSF12 (DR3) within CD95 + cells with higher relative expression of IL17A observed with CAF01, indicative of T_h_17 responses (Figure 3I, Figure 3—figure supplement 1G). Collectively, this gene-level analysis indicated strong engagement of T_h_1 molecular programs within LN CD4 T cells with MPLA.

### Phenotypically and functionally specialized GC T_fh_1 / T_fh_17 subsets elicited with HIV-1 Env formulated in CAF01 and MPLA+QS-21 adjuvants

**Figure 4.**
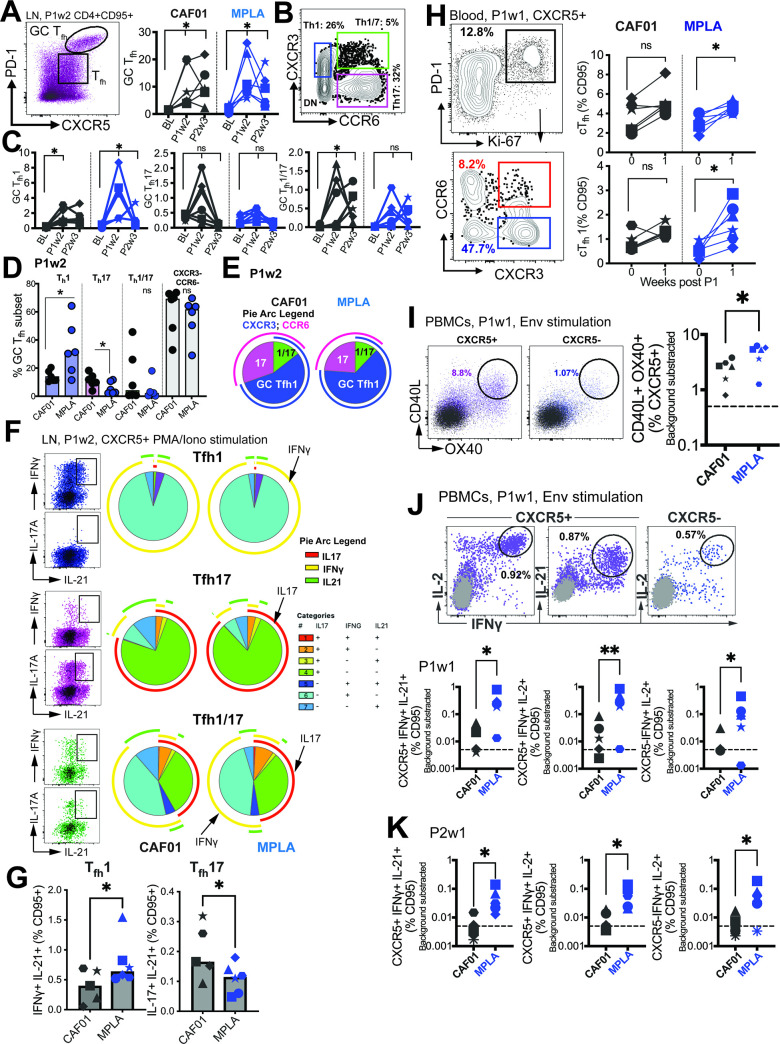
Phenotypically and functionally specialized GC T_fh_1 /T_fh_17 subsets elicited with HIV-1 Env Formulated in CAF01 and MPLA +QS-21 Adjuvants. (**A**) Representative flow cytometry plot illustrates GC T_fh_ cells and GC T_fh_ frequencies (%CD95) show in kinetic plot to right. (**B**) Flow cytometry plot shows identification of GC T_fh_1, T_fh_17, and T_fh_1/17 cells with (**C**) temporal kinetics expressed as %CD95+. Significance indicates differences in GC T_fh_ subsets at P1 and P2 relative to baseline. (**D**) Proportion of T_fh_1, T_fh_17, T_fh_1/7 and CXCR3-, CCR6- GC T_fh_ cells across CAF01 and MPLA at P1w2. (**E**) Boolean analysis (n=6 each group) shows proportion of T_h_1, T_h_17 and T_h_1/17 GC T_fh_ subsets. (**F–G**) Intracellular cytokine staining (ICS) analysis of T_h_1, T_fh_17, T_fh_1/17 subsets at P1w2. (**H**) Gating strategy (left) and frequencies (right) of circulating T_fh_ (cT_fh_) and cT_fh_1 cells in whole blood at P1w0 and P1w1. (**I**) Gating strategy (left) and frequencies (right) of activated (CD40L^+^OX40^+^) cT_fh_ (CXCR5^+^) and non-cT_fh_ CD4 T cells (CXCR5-) cells in PBMCs at P1w1. (**J**) ICS of cTfh and non-cTfh CD4 T cells following Env stimulation at P1w1 and P2w1. Data points show individual animals. Statistical analysis was performed using two-tailed Wilcoxon matched-pairs signed rank test (in panels **A**, **C, H**) or Mann-Whitney U test (in panels **D**; **G, I–K**); * p<0.05, **p<0.01.

After confirming the robust induction of GC responses by both vaccines and observing distinct molecular programs in CD4 T cells, we proceeded to investigate the phenotypic and functional characteristics of T_fh_ cells. We examined the GC T_fh_ cell subset characterized by the expression of CXCR5 and high levels of PD-1, using the gating strategy outlined in Figure 4—figure supplement 1A. Our analysis of the lymph nodes revealed an increase in the frequencies of GC T_fh_ cells following each protein boost in both groups compared to baseline. Importantly, there were no statistically significant differences observed between the two adjuvant platforms, as shown in Figure 4A. We further examined the frequencies of T_fh_ cells characterized by the expression of CXCR5 and PD-1 (CXCR5 +PD-1+), which also exhibited a significant increase following the first protein boost in both groups (Figure 4—figure supplement 1B).

**Figure 4—figure supplement 1.**
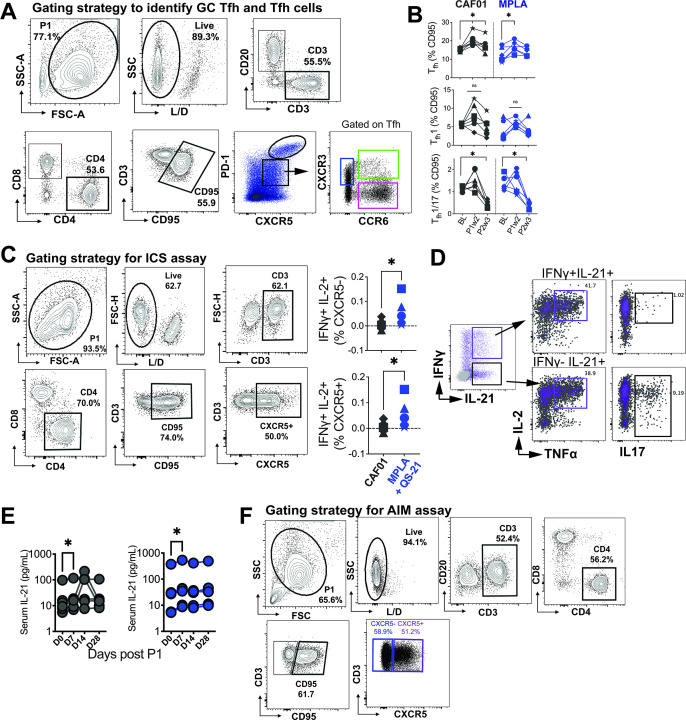
Gating strategy to identify GC Tfh and Tfh cells in lymph nodes. (**A**) Illustrates gating strategy to identify GC T_fh_ cells. (**B**) Frequencies of T_fh_ cells (CD4^+^CD95^+^PD-1^+^CXCR5^+^) (Top), T_fh_1 cells (CD4^+^CD95^+^PD-1^+^CXCR5^+^CXCR3^+^) (Middle), and T_fh_1/17 cells (CD4^+^CD95^+^PD-1^+^CXCR5^+^CXCR3^+^CCR6^+^) in lymph nodes. (**C**) Intracellular cytokine staining (ICS) to identify Env-specific T_fh_ cells. (**D**) Co-expression of IL-2 and TNFa in IFNG +versus IFNG- subsets producing IL-21. (**E**) Serum IL-21 post P1. (**F**) Gating of AIM assay.

To gain insights into the T_fh_ helper profiles within the GC, we assessed the expression of CXCR3 (T_fh_1) and CCR6 (T_fh_17) on the GC T_fh_ cells (flow plot in B). Both adjuvants induced GC T_fh_1 cells, as evidenced by the expression of CXCR3. However, a selective increase in GC T_fh_1/17 cells was observed with CAF01, while the frequencies of GC T_fh_17 cells did not show a significant increase following immunization (Figure 4C).

At P1w2, our investigation into the relative frequencies of polarized GC T_fh_ subsets revealed significant insights. We observed that most GC T_fh_ cells retained a non-polarized state (with respect to expression of CXCR3 and CCR6), with no substantial distinctions noted between the CAF01 and MPLA groups. Notably, the T_h_1 GC subset demonstrated a significant increase in the MPLA group, while the T_h_17 subset exhibited a higher prevalence in the CAF01 group (Figure 4D). This observation was further supported by Boolean analysis, which revealed a greater proportion of the T_h_17 subset associated with CAF01, and a higher proportion of the GC T_fh_1 subset with MPLA at P1w2, as illustrated in Figure 4E.

Following the observed induction of LN T_fh_1 responses, we quantified the Env-specific CD4 T cell response using intracellular cytokine staining (ICS). The analysis revealed higher relative frequencies of Env-specific T_fh_ cells and Env-specific CXCR5- CD4 T cells producing IFNγ with MPLA compared to CAF01 (Figure 4—figure supplement 1C). To obtain a more comprehensive understanding of the cytokine profile within the LN T_fh_1 and T_fh_17 subsets, we assessed cytokine production after PMA/Ionomycin stimulation (Figure 4F). By utilizing the discrete expression patterns of CXCR3 and CCR6 on CXCR5 +CD4 T cells in the LN, in conjunction with the canonical cytokines IFNγ (T_h_1), IL-17 (T_h_17), and IL-21 (T_fh_), we made several important observations. Firstly, we found that within the T_fh_1 subset, IL-21 +IFNγ+co-producing cells were more abundant, and their levels were significantly increased with MPLA stimulation (Figure 4G). Conversely, within the T_fh_17 subset, we observed that IL-21 +IL-17+co-producing cells were more abundant, and their levels were significantly higher in the CAF01 group. Notably, the T_fh_1/17 subset demonstrated the highest degree of polyfunctionality, as evidenced by the production of multiple cytokines.

We further analyzed the cytokine profiles by gating IFNγ and IL-21 co-producers versus IL-21 single producers. This analysis revealed that both subsets, T_fh_1 and T_fh_17, produced IL-2 and TNFα. However, IL-17 production was predominantly observed in IFNγ-negative, IL-21 + cells (Figure 4—figure supplement 1D). To assess systemic levels of IL-21, we measured serum IL-21. We found a significant induction of IL-21 in both vaccine groups at day 7 post-immunization, with no observable differences observed across the adjuvant platforms (Figure 4—figure supplement 1E). This suggests that the induction of IL-21 is a common feature of both vaccine formulations. Overall, these findings suggest that MPLA preferentially induces the production of IL-21 + IFNγ+ cells within the T_fh_1 subset, while CAF01 promotes the generation of IL-21 + IL-17+ cells within the T_fh_17 subset. Furthermore, the T_fh_1/17 subset exhibits the most diverse cytokine production profile among the subsets examined.

Next, we investigated whether the effects of MPLA versus CAF01 adjuvants were also evident in cTfh responses. We observed a significant increase in proliferating cTfh cells, with a predominant induction of cTfh1 cells, similar to the phenotype observed in the LN, when MPLA was used as the adjuvant (Figure 4H). In contrast, no evidence of a T_fh_17 or T_fh_1/17 bias in cTfh cells was observed with CAF01 (data not shown). To evaluate Env-specific T_fh_ cell abundance, we utilized the AIM assay (Figure 4—figure supplement 1F). The analysis showed a higher relative induction of Env-specific T_fh_ responses with MPLA at P1w1 but not P2w1 (Figure 4I), which was further corroborated by ICS assays (Figure 4J). The frequencies of CXCR5 + cells co-producing IL-2 and IFNγ, as well as cells co-producing IL-21 and IFNγ, indicated that higher numbers of IFNγ+Env-specific cTfh cells were induced with MPLA at P1w1 and P2w1 (Figure 4K). Therefore, formulation of gp140 with MPLA induced strong T_h_1-polarized GC T_fh_ responses, characterized by a higher magnitude of IFNγ+anti Env T_fh_ cells.

### Induction of persistent anti-Env IgG antibodies with HIV-1 Env/MPLA+QS-21

**Figure 5.**
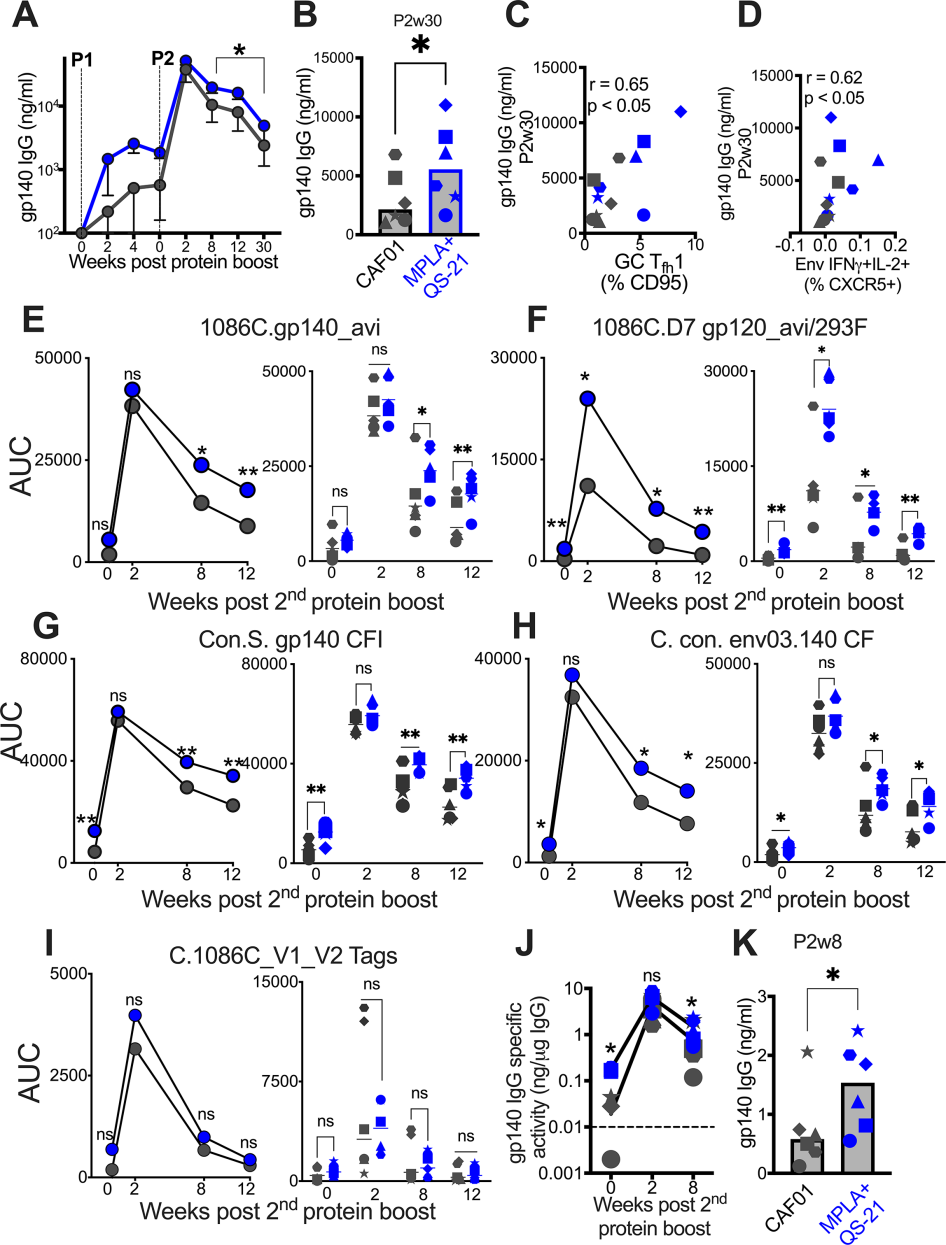
Induction of persistent anti-Env IgG antibodies with HIV-1 Env/MPLA +QS-21. (**A**) Kinetics of HIV-1 Env IgG post protein boost (P1 and P2) across vaccine regimens measured by ELISA against C. 1086 gp140. (**B**) Serum gp140 IgG week 30 post P2w3. (**C**) GC T_fh_1 cells correlate with gp140 IgG at week 30. (**D**) Env-specific (IFN-g^+^IL-2^+^) T_fh_ cells correlate with gp140 IgG at week 30. (**E–I**) Area under the curve (AUC) values of IgG temporally shown by antigens, as indicated. (**J**) Temporal and (**K**) week 8 measures of gp140-specific IgG levels relative to total IgG in rectal secretions. Data points show individual animals. Statistical analysis was performed using Mann-Whitney U test (in panels **A-B**, **E–K**), or two-tailed Spearman rank correlation test (**C–D**); * p<0.05, **p<0.01.

We next sought to determine whether induction of higher frequencies of Env-specific T_fh_1 cells with MPLA could effectively enhance B cell proliferation and differentiation, in turn, promoting the development of Env antibodies with heightened affinity and increased durability. To this end, we performed a wide repertoire of quantitative and functional assays to assess the magnitude, durability, affinity, avidity, reactivity to Group M consensus protein, and effector functionality of vaccine-induced humoral responses to Env. Longitudinal measures of binding antibody concentrations in sera against C.1086 Env showed rapid and equivalent recall in all animal’s post P1, with antibodies reaching peak levels after the second protein boost. While antibody kinetics were comparable across vaccine groups, significantly higher antibody magnitude was elicited by MPLA at week 8 post second boost and until 30 weeks after final immunization (Figure 5A). At this protracted memory time point, serum antibody concentrations were, on average, 2.5-fold higher with MPLA (median [ng/ml]; MPLA, 5566 versus CAF01, 2155) (Figure 5B). To assess CD4 correlates of antibody durability, we asked whether the magnitude of vaccine-elicited GC T_fh_ subsets after each boost predicted antibody concentrations at week 30. Frequencies of GC T_fh_ cells (at P1w2) were positively associated with antibodies at week 30 (*r*=0.6, p<0.05, data not shown). Two additional key cellular determinants of antibody durability emerged from our analysis: GC T_fh_1 cell magnitude (p<0.05, *r*=0.65) (Figure 5C) and frequencies of IFNγ+IL-2+Env-specific LN T_fh_ cells (p<0.05, *r*=0.62; Figure 5D). These data in concert with IFNγ-regulated molecular programs induced in LN CD4 T cells support a role for GC T_fh_1 induction in antibody persistence.

Using a binding antibody multiplex assay (BAMA), we further confirmed that stronger memory responses were elicited by MPLA, evidenced by higher median serum IgG antibody concentrations to C.1086 gp140 and gp120 antigens measured as area-under-the curve (AUC) over time (Figure 5E–F). Thus, utilizing two orthogonal approaches, the data showed significantly higher and protracted serum antibody responses against autologous Env with MPLA, indicative of efficient T_fh_ cell mediated B cell recruitment into the long-lived plasma cell pool.

We next sought to understand if induction of a T_fh_1 profile was also associated with improved Env reactivity to Group M consensus proteins. Therefore, we tested serum reactivity against an antigen panel comprising of consensus gp140 proteins and found that antibodies with higher cross-clade reactivity were induced with MPLA relative to CAF01 (Figure 5G–H). We extended these analyses to assess reactivity to V1V2 loops of 1086 C and noted that antibodies with significantly higher specificity to these important regions within Env were induced in both vaccine regimens (Figure 5I).

Having established induction of robust serum Env antibodies with MPLA, we next determined whether Env-specific IgG reactivity would also be higher within rectal secretions. After normalization for total IgG levels within rectal secretions, the data showed that Env antibodies were effectively recalled in most animals at week 2 post second protein boost and persisted in all animals 8 weeks after final immunization. However, akin to the systemic compartment, gp140 IgG in rectal secretions were significantly higher with MPLA at memory time point (Figure 5J), with sixfold higher levels in MPLA relative to CAF01 at week 8 after final immunization (Figure 5K). In both vaccine regimens, the induction of Env-IgA antibodies was poor, preventing a quantitative assessment of IgA responses in secretions.

### Induction of IgG1 subclass antibodies with greater effector functions with HIV-1 Env/MPLA+QS-21

Since antibody effector functions, facilitated by interaction of Ig constant region with cognate Fc receptors on innate cells, mediate protection from acquisition and viral control (Carpenter and Ackerman, 2020), we assessed the IgG subclass profile at week 2 post second protein boost. Both CAF01 and MPLA +QS-21 induced Env-specific IgG1, and modest levels of IgG2, and IgG4 isotypes with higher IgG1 in all animals, with exception of one animal with higher IgG4 (Figure 6—figure supplement 1).

**Figure 6.**
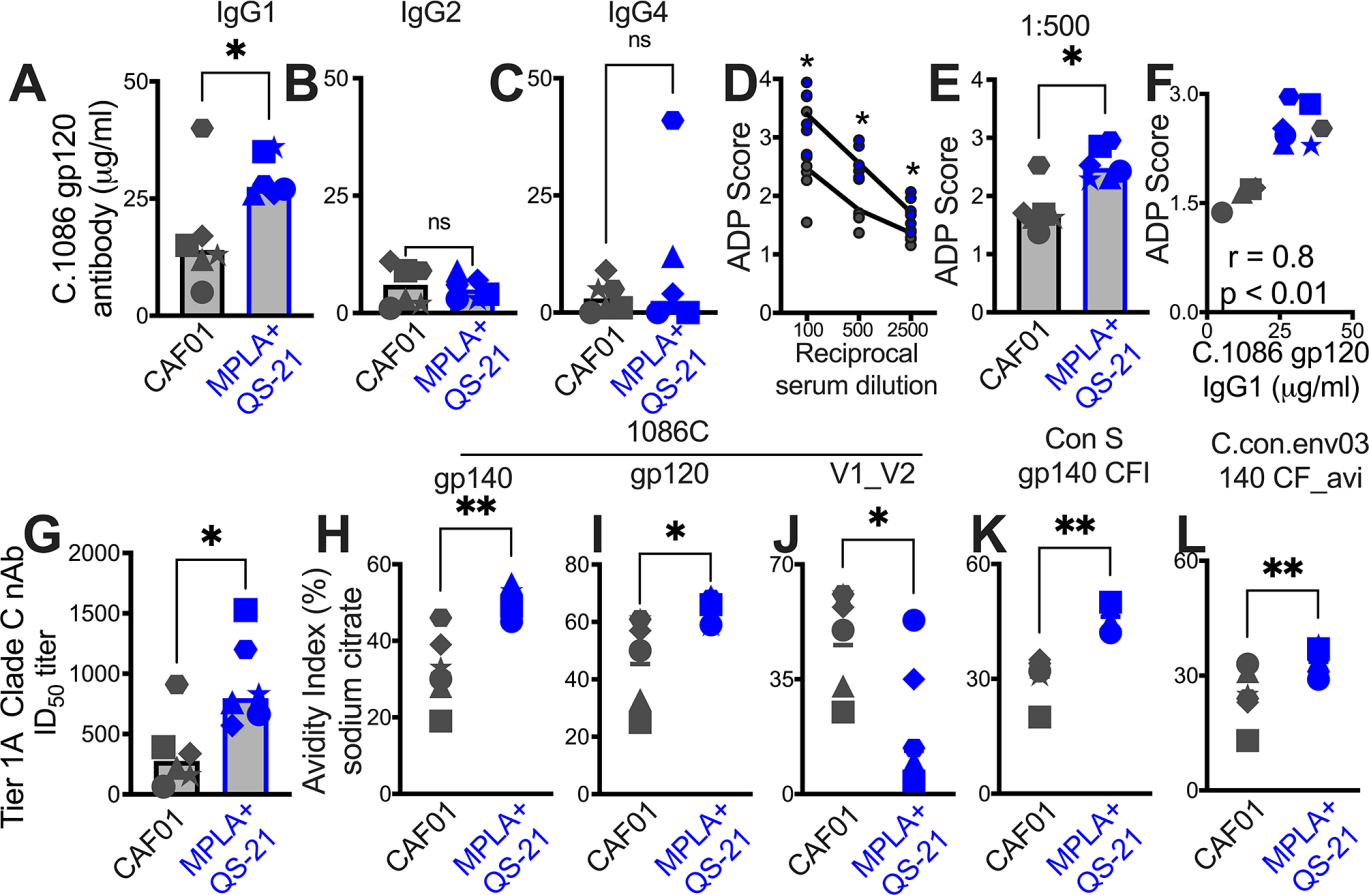
Induction of IgG1 subclass antibodies with greater effector functions with HIV-1 Env/MPLA +QS-21. Serum C. 1086 gp120-specific (**A**) IgG1, (**B**) IgG2 (**C**) IgG4 antibodies at P2w2. (**D–E**) Antibody-dependent phagocytosis (ADP) score at P2w8. (**F**) C. 1086 gp120-specific IgG1 correlates with ADP score. (**G**) Infectious dose 50% (ID50) titers to Tier 1 A Clade C MW965.26 HIV-1 isolate at P2w2. (**H–L**) Avidity index (with sodium citrate) across vaccine regimens against specific antigens at P2w8. Data points show individual animals. Statistical analysis was performed using two-tailed Mann-Whitney U test (in panels **A-E**; **G–L**), or two-tailed Spearman rank correlation test (**F**); * p<0.05, **p<0.01.

Significantly higher IgG1 was induced by MPLA relative to CAF01 (Figure 6A), while IgG2 and IgG4 antibody subclasses were comparably induced (Figure 6B–C). The improved IgG1 subtype antibody responses with MPLA prompted us to determine whether a corresponding increase in antibody effector functions might also be observed. Measurement of antibody-dependent phagocytosis (ADP) suggested enhanced effector functions with MPLA (Figure 6D–E) which strongly correlated with Env IgG1 but not IgG2 or IgG4 (Figure 6F).

**Figure 6—figure supplement 1.**
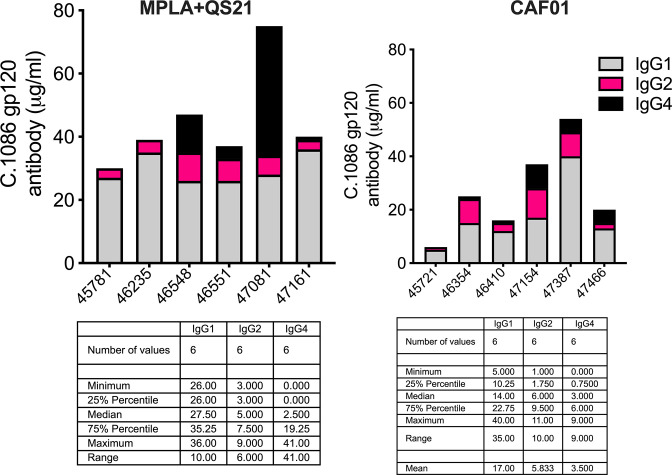
Serum C. 1086 gp120-specific IgG1 (grey), IgG2 (pink), and IgG4 (black) in animals (x-axis – animal IDs) P2w3.

Finally, we observed higher levels of neutralizing antibodies against the tier 1A MW965.26 at peak (2 weeks post second protein boost) with MPLA relative to the CAF01 platform (Figure 6G). Antibody avidity measurements revealed relatively higher avidity with MPLA against autologous Env and to consensus Env proteins but not V1V2 loops (Figure 6H–L). Collectively, these findings provide evidence for significantly improved antibody quality associated with induction of GC T_fh_1 cells.

### Differentiation of GC T_fh_ subsets initiated during the DNA priming phase

**Figure 7.**
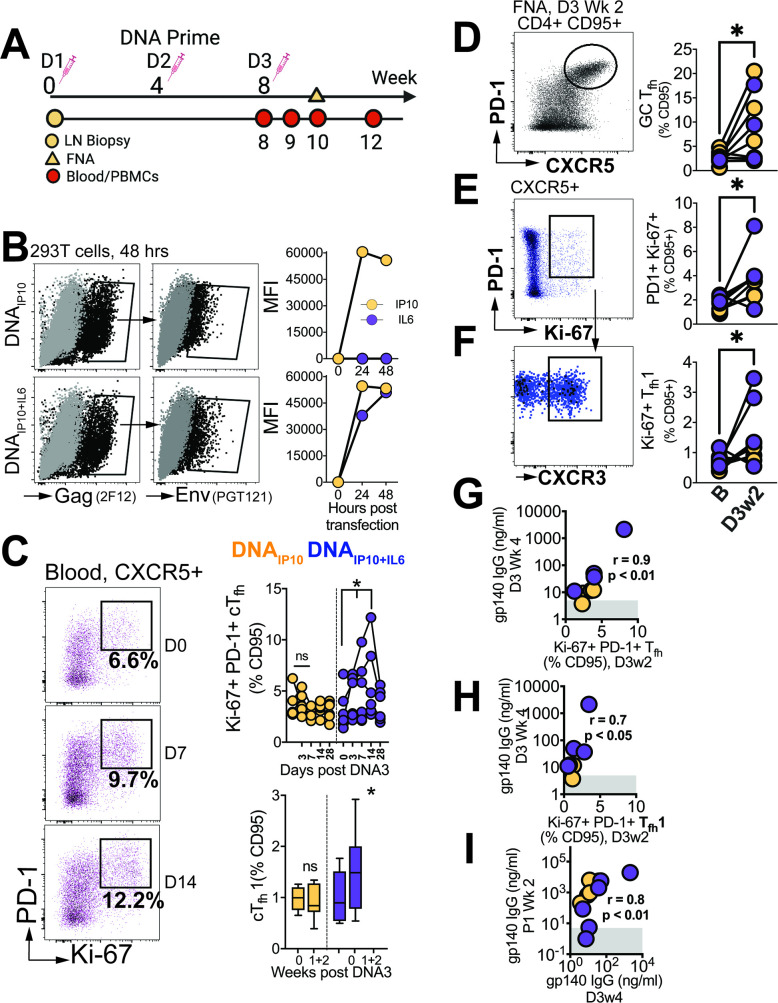
Differentiation of GC T_fh_ Subsets initiated during the DNA priming phase. (**A**) Experimental design of DNA immunization phase; FNA, lymph node fine needle aspirates. (**B**) Intracellular expression of Gag (2F12), surface expression of HIV-1 Env (PGT121) and IP10 and IL6 in supernatants of transfected 293T cells. (**C**) Flow cytometry plots show activated (PD-1^+^Ki-67^+^) cTfh cells, frequencies post DNA3 (right top); frequencies of cTfh1 cells (right bottom). (**D**) GC T_fh_ cells, (**E**) Ki-67^+^ PD1^+^ cells of CXCR5 +subset in lymph node. (**F**) CXCR3 +subset of Ki-67^+^ PD-1^+^ CXCR5^+^ subset. (**G**) Ki-67 +PD-1+T_fh_ in LN correlate with gp140 IgG at week 4 post DNA3. (**H**) Ki-67 +PD-1+T_fh_1 in LN correlate with gp140 IgG at week 4 post DNA. (**I**) gp140 IgG at week 2 post P1 correlates with gp140 IgG at week 4 post DNA3. Data points show individual animals. Statistical analysis was performed using one-tailed Wilcoxon matched-pairs signed rank test (in panels C-F), or Spearman rank correlation test (**G–I**); * p<0.05.

Finally, we sought to determine the degree to which T_fh_ responses elicited during the priming phase influenced antibody responses post first protein boost. To this end, we analyzed blood samples at weeks 0, 1, 2, and 4, while also evaluating FNA of the draining LN at week 2 after the 3rd DNA prime (Figure 7A).

**Figure 7—figure supplement 1.**
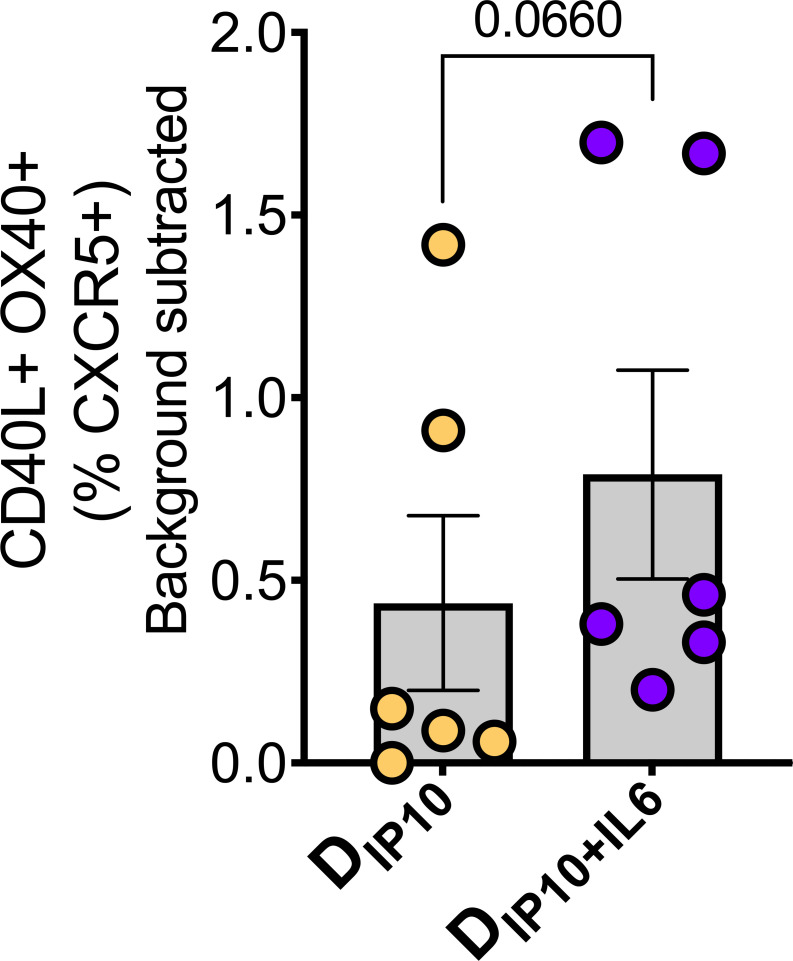
Frequencies of OX40^+^CD40L^+^ cTfh cells stimulated ex vivo with Gag and Env.

Animals were immunized with C.1086 DNA expressing IP10 (DNA_IP10_) which primes stronger GC T_fh_ responses relative to DNA-alone (Verma et al., 2019). To further enhance GC T_fh_ responses, beyond that induced by DNA_IP10_, we engineered DNA_IP10_ to co-express IL-6, a key cytokine promoting T_fh_ differentiation. Our vector achieved coordinate expression of Gag and Env antigens together with IP10 +IL6 as confirmed in transfected 293T cells (Figure 7B). Following three rounds of intradermal immunization with DNA_IP10_ or DNA_IP10+IL6_, we assessed proliferating cTfh cells (Ki-67^+^ PD-1^+^ of CXCR5 +CD4 T cells in blood, Figure 7C). We observed a significant increase at weeks 1 and 2 with DNA_IP10+IL6_ but not DNA_IP10_ indicating that IP10 +IL6 incorporation promoted T_fh_ differentiation. Additionally, T_fh_ induction was accompanied by notable T_fh_1 skewing within proliferating cTfh cells (median cTfh1 at day 0 (% CD95), 0.89% vs 1.48% at day 7 and 14). Measurement of antigen-specific responses by AIM assay demonstrated induction of Env and Gag-specific cTfh cells with a trend for higher responses elicited by DNA_IP10+IL6_ (median, 0.42%, IQR 0.3–1.7%) relative to D_IP10_ (median, 0.12%; IQR: 0.04–1.4%, Figure 7—figure supplement 1).

Based on the transient accumulation of cTfh cells in the blood, we further explored GC T_fh_ responses. Out of 12 collected FNAs, 8 were successful (four per vaccine group), and demonstrated significant increase in GC T_fh_ frequencies following vaccination (Figure 7D). Gating on Ki-67 + cells encompassing both CXCR5 + PD-1+and PD-1 ++ subsets, an increase in activated T_fh_ cells within the LN was observed (Figure 7E), accompanied by a corresponding increase in the T_fh_1 subset (Figure 7F). Altogether, these data demonstrate induction of GC T_fh_1 responses during the priming phase post immunization with DNA_IP10_ or DNA_IP10+IL6_.

Measurement of C.1086 Env antibodies in serum revealed induction of antibody responses after the 3rd prime in 8/12 animals, with comparable kinetics across vaccine groups. Notably, antibody concentrations 4 weeks post 3rd DNA prime strongly correlated with both the frequencies of proliferating T_fh_ cells (Ki-67 +PD-1+CXCR5+) within the LN and with the proliferating T_fh_1 (Ki-67 +PD-1+CXCR5+CXCR3+) subset at 2 weeks post 3rd DNA prime (Figure 7G–H). Moreover, the strong association of antibody concentrations after the 3rd DNA prime with peak antibodies following the first protein boost (Figure 7I) indicated efficient recall of Env-specific memory B cells induced during the prime. Overall, the data show that stimulation of T_fh_1 cells during the DNA priming stage is closely linked with robust antibodies post boost.

In summary, our results strongly support maximizing GC T_fh_1 responses during both the prime and boost phases as a strategy to generate potent and durable humoral immunity. These findings have significant implications for the development of more effective vaccination strategies aimed at eliciting robust and long-lasting immune responses.

## Discussion

T_fh_ cells are fundamental in establishing long-lasting humoral immunity with vaccination. The dynamic interplay between innate immune activation and the ensuing inflammatory response directs the magnitude and composition of T_fh_ cell populations, ultimately shaping quantitative and qualitative features of humoral memory (Hirota et al., 2013; Barbet et al., 2018; Olatunde et al., 2021). Here we show that maximizing induction of CXCR3 +T_fh_1 cells correlates with serum Env antibodies which exhibit higher persistence. Our studies with LAMV revealed the robust induction of cTfh1 and GC T_fh_1 responses, along with a moderate induction of T_fh_17 and T_fh_1/17 cells. These findings led us to compare the adjuvants MPLA +QS-21 and CAF01, known to elicit T_h_1 and mixed T_h_1/17 responses (Christensen et al., 2019), (Pedersen et al., 2018) respectively, to determine the optimal strategy for maximizing Env antibody persistence. Through proteomic and transcriptomic analysis of lymph nodes, we uncovered evidence of robust GC responses induced by both vaccine adjuvants following a DNA prime. Moreover, we observed notable skewing of T_fh_ responses, which corresponded with the elicitation of distinct immunogenicity profiles. Interesting, although peak antibody responses following each protein boost were similar across the vaccine platforms, our investigation revealed a notable distinction in antibody persistence. Specifically, the MPLA +QS-21 regimen exhibited higher antibody levels at week 8, 12, and 30, suggestive of enhanced support from T_fh_ cells in promoting plasma cell differentiation. Moreover, T_fh_1 cell induction during the DNA priming phase was linked to effective boosting upon subsequent protein immunization. These findings highlight the critical role of T_fh_1 cells in driving the longevity of antibody responses and emphasize the potential of stimulating T_fh_1 cells during the prime and boost to elicit durable humoral immunity against HIV Env.

A significant finding in RV144 vaccine recipients was the correlation between Env-specific circulating CD4 T cell subsets co-producing IFNγ, TNFα, CD40L, and IL4, and a reduced risk of HIV acquisition [40]. This correlation suggests that productive GC responses elicited by the vaccine fostered protective humoral immunity (Lin et al., 2015). More recently, follow up analysis of this population in n=6 subjects immunized with the RV144 vaccine regimen in South Africa (HVTN 097) reported a pT_h_2 biased transcriptional profile based on observed IL4 and IL13 transcripts (Cohen et al., 2022). However, it is important to note that in addition to T_h_2 cells, CD4 T_fh_ cells also produce IL-4 in a SLAM/SAP-dependent manner (McCausland et al., 2007) and T_h_1 cells can produce IL-13 in the presence of IL18 (Hayashi et al., 2007). These findings indicate the need for utilizing multiple immune parameters when categorizing CD4 T cell help and underscore the importance of studying both lymph node and peripheral blood CD4 T_fh_ cells to fully understand their respective roles in fostering Env antibody responses.

In the context of vaccination, our observed association between T_fh_1 cells and persistent antibody responses raises the possibly that higher relative production of IL-21 and CD40L may support the proliferation of GC B cells in concert with IFNγ-mediated B cell proliferation. This contention is supported by the observation of higher relative Ki-67 protein levels in the MPLA group at week 2 of the first protein boost. In the context of a T_h_1 driven GC response rich in CXCL chemokines CXCL9, 10, and 11; CXCR3 expression on T_fh_ cells may furthermore enable proximity and interaction with GC B cells. CAF01 is composed of cationic liposomal vesicle (dimethyldioctadecylammonium, DDA) together with a synthetic analog of a mycobacterial cell wall (trehalose dibehenate, TDB), a potent activator of macrophages and dendritic cells through the macrophage inducible Ca2^+^-dependent lectin receptor Mincle, a pattern recognition receptor recognizing bacterial lipids (Davidsen et al., 2005; Martínez-López et al., 2019). CAF01 elicits CD4 T_h_1 responses while concurrently driving strong T_h_17 responses to a mycobacterium TB subunit vaccine via induction of proinflammatory cytokines IL-1β, IL-6, and TNF-α (Werninghaus et al., 2009). We observed that CAF01 was effective in inducing robust cellular responses, which were skewed towards a T_h_1/T_h_17 profile within GCs. It is possible that employing the CAF06 platform which incorporates MPL into the bilayer of DDA/TDB liposomes may drive stronger T_h_1 responses together with T_h_17 cells thereby enhancing protective immunity by eliciting strong humoral immune responses.

The present studies are the first to our knowledge to assess immunological responses across HIV vaccine platforms with MPLA and CAF01-based adjuvants and strongly support GC T_fh_1 cell induction for enhancing Env antibody responses. However, our study has several limitations. First, priming modalities across MPLA and CAF01 were not identical. Animals boosted with MPLA were primed with DNA_IP10_, whereas CAF01 boosted animals received the DNA_IP10+IL6_ prime. Given the stronger immunogenicity of DNA_IP10+IL6_, it is possible that our studies underestimate immune recall potential of MPLA relative to CAF01. Second, 5/6 animals in each vaccine group were males and therefore our findings do not capture possible variability in vaccine response between sexes. Because females develop higher antibody responses than males, it is important to test our hypothesis in animals of both sexes (Fischinger et al., 2019). Third, the interplay of innate immune cells and the follicular dendritic cell network in regulation of T_fh_ help for antibody responses is an important consideration for future studies. In this context, conducting mechanistic studies involving cytokine blockade to assess its impact on T_fh_ differentiation in vivo is essential for establishing the significance of adjuvants in shaping humoral immunity. Finally, we did not assess vaccine efficacy and therefore whether GC T_fh_1 cells constitute an important correlate of protection from acquisition remains an important unanswered question.

In summary, our findings demonstrate significant skewing of the GC T_fh_ response by adjuvants inducing distinct inflammatory responses. We further demonstrate that induction of T_fh_1 cells results in superior persistence of Env antibodies indicating that strategies to harness this CD4 subset may promote protective humoral immunity against HIV.

## Materials and methods

### Rhesus macaques

For LAMV studies, sixteen female colony-bred Indian origin rhesus macaques (*Macaca mulatta*) were utilized. Animals were dichotomized by age into two groups; young [n=8, mean = 4 years] and aged [n=8, mean = 16 years]. Young adults with a higher baseline weight [mean = 5.9 kg] were selected for the cohort to closer match the aged animals in size [mean = 8.6 kg]. For the HIV vaccine study, 12 adult (10 males and 2 females) Indian origin rhesus macaques (*Macaca mulatta*) were utilized. At study initiation, animals were 3.4–5.6 years of age with a median weight of 6.8 kg. Animals in both studies were SIV negative (SIV-), simian T-cell leukemia virus negative (STLV-), and simian retrovirus negative (SRV-); and had no history of dietary, pharmacological, or surgical manipulation. All animals were bred and housed at the California National Primate Research Center (CNPRC) in accordance with the American Association for Accreditation of Laboratory Animal Care (AAALAC) guidelines. All studies were approved by the University of California, Davis Institutional Animal Care and Use Committee (IACUC).

### LAMV immunization

The animals received an IM booster vaccination of canine distemper-measles vaccine (Vanguard).

### HIV-1 immunization

Animals were immunized with DNA vaccines intradermally in both thighs at weeks 0, 4, and 8. At each prime, animals received 4 mg of the pGA2/JS2 plasmid DNA encoding either SHIV C.1086 T/F Env +rhesus interferon-induced protein (IP)–10 (Group 1; n=6) or SHIV C.1086 T/F Env +rhesus interferon-induced protein (IP)–10+rhesus interleukin (IL)–6 (Group 2; n=6). Subsequent protein boosters were administered at weeks 12 and 20 with Group 1 animals receiving 50 μg C.ZA 1197 MB gp140 protein adjuvanted with MPLA +QS-21 and Group 2 animals receiving 50 μg C.ZA 1197 MB gp140 protein adjuvanted with CAF01 delivered in a 175 μL volume subcutaneously into each thigh.

### Cationic adjuvant formulation 01 (CAF01)

CAF01 was generously provided by Statens Serum Institut, Denmark. Admixing with protein was performed by addition of 25 μg of C.ZA 1197 MB gp140 protein (Immune Technology, USA) to 250 µL of CAF01 (625 μg DDA +125 μg TDB) adjuvant formulation. The solution was vortexed vigorously for 15–30 s followed by visual inspection. Remaining protein was added in 10 μg increments with intermittent vortexing until a final amount of 100 μg gp140 was achieved.

### Monophosphoryl lipid A (MPLA)

Synthetic MPLA was purchased from InvivoGen, USA. 100 μg C.ZA 1197 MB gp140 protein was dissolved in a solution of 100 μg MPLA with 50 μg QS-21 saponin [Desert King International, USA] (2:2:1) in PBS.

### Specimen collection and processing

Lymph node (LN) biopsies were obtained at baseline (week 0), week 14 (week 2 post first protein boost) and week 23 (week 3 post second protein boost) and processed as described previously (Verma et al., 2019). Isolated cells were washed in complete media, counted, and cryopreserved until subsequent analysis. Two weeks after the 3rd DNA immunization, fine needle aspirates of LN were obtained using a 22-gauge needle, as previously described (Verma et al., 2019). PBMCs were isolated from whole blood collected in CPT vacutainer tubes at weeks 0, 4, 9, 10, 13, 21, and 32. Serum was collected from animals at weeks 0, 4, 8, 8+day 3, 9,10, 12, 13, 14, 16, 20, 20+day 3, 21, 22, 23, 28, 32, 50 timepoints and stored at –80 °C until subsequent analysis. Rectal secretions were collected for assessment of mucosal antibody production using premoistened Weck-Cel sponges as previously described (Verma et al., 2019).

### Activation induced marker and intracellular cytokine staining assay

Detection of antigen specific CD4 T cells detection was quantified by activation induced marker (AIM) and intracellular cytokine staining (ICS). PBMCs/LN cells were stimulated with overlapping peptide pools of HIV consensus C and HIV-1 C.1086 Env gp140C protein (NIH AIDS Reagent Program) in AIM/R10 media in the presence of 0.2 μg CD28/49d co-stimulatory antibodies (BD) per test. As a positive control, cells were stimulated with 1 X Cell Stimulation Cocktail (PMA and ionomycin) (eBioscience, USA). Unstimulated controls were treated with volume-controlled DMSO (Sigma-Aldrich). Tubes were incubated in 5% CO_2_ at 37 °C overnight for AIM assay. For ICS assay, after 1 hr of stimulations, protein transport inhibitors 2 μl/mL GolgiPlug (Brefeldin A) and 1.3 μl/mL GolgiStop (Monensin) (BD, Biosciences, USA) were added to the tubes for 8 hr at 37 °C, 5% CO_2_. Following stimulation, the cells were stained for AIM and ICS surface markers (see Table 1). Cells were then fixed with cytofix/cytoperm for 10 min at 4 °C, permeabilized with 1 X Perm wash buffer (BD, Biosciences, USA), and stained for intracellular markers (see Table 1) for 45 min. Cells were then washed and acquired the same day on a BD FACS Symphony.

**Table 1.** Flow cytometry antibodies.

Antibody name	Panel	Vendor	Catalog number/Identifier
Mouse anti-human CD3 (Clone SP34-2)	T_FH_/AIM/ICS	BD Biosciences	Cat#557917; RRID: AB_396938
Mouse anti-human CD4 (Clone L200)	T_FH_/AIM/ICS	BD Biosciences	Cat#563737; RRID: AB2687486
Mouse anti-human CD8 (Clone SK-1)	T_FH_/AIM/ICS	BD Biosciences	Cat#564913; RRID: AB_2833078
Mouse anti-human CD14 (Clone MSE2)	T_FH_ Panel	BioLegend	Cat#301822; RRID: AB_493747
Mouse anti-human CD16 (Clone 3G8)	T_FH_ Panel	BD Biosciences	Cat#563172; RRID: AB_2744297
Mouse anti-human CD20 (Clone 2H7)	T_FH_ Panel	BioLegend	Cat#302314; RRID: AB_314262
Mouse anti-human CD69 (Clone FN50)	T_FH_ Panel	BioLegend	Cat#310944; RRID: AB_2566466
Mouse anti-human CD95 (Clone DX2)	T_FH_/AIM/ICS	BioLegend	Cat#564710; RRID: AB_2738907
Mouse anti-human CXCR3 (CD183) (Clone 1C6)	T_FH_ Panel	BD Biosciences	Cat# 550967; RRID: AB_398481
Mouse anti-human CXCR5 (CD185) (Clone MU5UBEE)	T_FH_/AIM/ICS	eBioscience	Cat#12-9185-42; RRID: AB_11219877
Mouse anti-human CCR6 (CD196) (Clone G034E3)	T_FH_ Panel	BioLegend	Cat#353430; RRID: AB_2564233
Armenian Hamster anti- ICOS (CD278) (Clone C396.4A)	T_FH_ Panel	BioLegend	Cat#313534; RRID: AB_2629729
PECy7 anti-human PD1 (CD279) (Clone EH12.2H8)	T_FH_ Panel	BioLegend	Cat# 329918, RRID: AB_2159324
Mouse anti-human Bcl-6 (Clone K112-91)	T_FH_ Panel	BD Biosciences	Cat# 563581
Mouse anti-Ki-67 (Clone B56)	T_FH_ Panel	BD Biosciences	Cat#558616; RRID: AB_10611866
Mouse anti-human CD25 (Clone BC96)	AIM assay	eBioscience	Cat#**53-0259-42** RRID: AB_2043827
Mouse anti-human CD134 (OX-40) (Clone L106)	AIM assay	BD Biosciences	Cat#744746; RRID: AB_2742454
Mouse anti-human CD137 (4-1BB) (Clone 4B4-1)	AIM assay	BioLegend	Cat# 309826; RRID: AB_2566260
Mouse anti-human CD154 (CD40L) (Clone 24–31)	AIM assay	eBioscience	Cat#17154842 RRID:AB_1582215
Mouse anti-human TNF-α (Clone Mab11)	ICS assay	BioLegend	Cat# 502906; RRID: AB_315258
Mouse anti-human IFNγ (Clone B27)	ICS assay	BioLegend	Cat# 506518; RRID: AB_2123321
Mouse anti-human IL2 (Clone MO1-17H12)	ICS assay	BioLegend	Cat# 500344; RRID: AB_2564091
Mouse anti-human IL-17 (Clone eBio64DEC17)	ICS assay	eBioscience	Cat# 48-7179-42; RRID: AB_10853643
Mouse anti-human IL-21 (Clone 3A3-N2.1)	ICS assay	BD Biosciences	Cat# 560493; RRID: AB_1645421
APC-Cy7 live/dead		Life Technologies	Cat#L34976
BV510 live/dead		Life Technologies	Cat#L34966

### T_fh_ cell staining by flow cytometry and cell sorting

T_fh_ cell staining was performed on whole blood and LN cells as previously described (Verma et al., 2019). Samples were acquired on BD FACS Symphony with FACS Diva version 8.0.1 software and data were analyzed using FlowJo (Versions 9 and 10). For cell sorting, cryopreserved cells were enriched by NHP CD4 isolation kit (Miltenyi Biotec,USA) and stained with CD3, CD4, CXCR5, CD95, and live/dead in complete media (incubated for 1 hr at 4 °C on a shaker). Stained cells were washed twice with 5 mL RPMI plain media (Gibco, USA) and resuspended in 0.5 mL of sorting buffer containing 2% FBS +PBS. The cells were then filtered through a 40 μM strainer into 5 mL sterile FACS tube (Blue cap tube). Cell sorting was performed using a BD FACSAria III. Naive, and CD95 +CD4 T cell populations were collected in complete media supplemented with 20% FBS. Sorted cells were stimulated with overlapping peptide pools of HIV consensus C and HIV-1 C.1086 Env gp140C protein (NIH AIDS Reagent Program) in R10 media in the presence of 0.2 μg/mL CD28/49d co-stimulatory antibodies (BD) for 14 hr. The culture supernatant and stimulated cells were collected and stored at –80 °C for subsequent analysis.

### Serum IL-21 ELISA

Serum IL-21 cytokine was quantified using an IL-21 ELISA Development kit (Novus Bio, USA) in accordance with manufacturer’s protocol. Briefly, the capture mAb (MT216G) was diluted to 2 μg/mL in PBS, pH 7.4 and added (100 μL/well) to 96-well microtiter plates with high binding capacity (Thermo Fisher, USA) and incubated overnight at 4 °C. The next day, plates were aspirated, and wells blocked with 200 μL/well of PBS with 0.05% Tween 20 and 0.1% BSA (incubation buffer). After 1 hr of incubation, plates were washed five times with PBS containing 0.05% Tween 20 (300μL/well). Samples or working standards were added at 100 µL/well and incubated for 2 hr at room temperature. After washing, plates were incubated for 1 hr with 100 μL/well of detection mAb diluted to 1 μg/mL in incubation buffer. Streptavidin-HRP conjugates were diluted 1: 1000 in incubation buffer and added to the plates at 100 µL/well for 1 hr followed by washing. Plates were washed and then developed with TMB substrate (Thermo Fisher, USA), and the reaction quenched with 0.2 M H_2_SO_4_ (Sigma, USA). Absorbance was recorded using a Spectramax 5 plate reader (Molecular Devices) at 450 nm with a reference filter at 570 nm within 15 min. The concentration of IL-21 in serum was calculated based on an IL-21 standard curve using SoftMax Pro.

### RNA sequencing and bioinformatics

RNA was extracted from sorted naive, and CD95 +CD4 T cells using a RNeasy plus mini kit (QIAGEN) (Table 2). Isolated RNA sample quality was assessed using a BioAnalyzer RNA pico assay (Agilent Technologies Inc, California, USA) and quantified by Qubit 2.0 RNA HS assay (Thermo Fisher, Massachusetts, USA). Library construction was performed based on manufacturer’s recommendation for the SMART-Seq v4 Ultra Low Input RNA Kit (Takara Bio USA Inc, California, USA) followed by the Nextera XT DNA Library Prep Kit (Illumina, California, USA). Final library quantity was measured using the KAPA SYBR FAST qPCR and library quality evaluated using a TapeStation D1000 ScreenTape (Agilent Technologies, CA, USA). Final library size was about 450 bp with an insert size of about 300 bp. Illumina 8-nt dual-indices were used. Equimolar pooling of libraries was performed based on QC values and sequenced on an Illumina NovaSeq S4 (Illumina, California, USA) with a read length configuration of 150 PE for 40 M PE reads per sample (20 M in each direction). Reference rhesus macaque genome (*Macaca_mulatta* _GCF_003339765.1_Mmul_10) and gene model annotation files were downloaded directly from the genome website. Index of the reference genome was built using Hisat2 v2.0.5 and paired-end clean reads were aligned to the reference genome using Hisat2 v2.0.5. The mapped reads of each sample were assembled by StringTie (v1.3.3b) using a reference-based approach.

**Table 2.** Samples for RNA seq and Spatial profiling.

S.No	Animals ID	Vaccine group	Lymph node collection time points and cells used for		
			Baseline (WK0)	Week2 post 1st protein boost	Week3 post 2nd protein boost
1	**47161**	MPLA +QS-21	RNA seq	RNA seq	RNA seq
2	**45781**	MPLA +QS-21	Spatial profiling	Spatial profiling	Spatial profiling
3	**46235**	MPLA +QS-21	Spatial profiling	Spatial profiling	Spatial profiling
4	**46551**	MPLA +QS-21	Spatial profiling	Spatial profiling	Spatial profiling
5	**46548**	MPLA +QS-21	RNA seq	RNA seq	RNA seq
6	**47081**	MPLA +QS-21	RNA seq	RNA seq	RNA seq
7	**45721**	CAF01	RNA seq +Spatial profiling	RNA seq +Spatial profiling	RNA seq +Spatial profiling
8	**47154**	CAF01	NA	NA	NA
9	**46410**	CAF01	Spatial profiling	Spatial profiling	Spatial profiling
10	**46354**	CAF01	RNA seq	RNA seq	RNA seq
11	**47466**	CAF01	RNA seq	RNA seq	RNA seq
12	**47387**	CAF01	Spatial profiling	Spatial profiling	Spatial profiling
					

The quality of the raw RNA-seq data was assessed using FastQC and poor-quality ends were trimmed (Trimgalore). High-quality sequences were aligned against the *Macaca mulatta* reference genome using STAR aligner v2.7.9, and counts were generated using featureCount (Dobin et al., 2013). A list of differentially expressed genes (DEGs) were generated using DESeq2 based on the negative binomial distribution (Love et al., 2014). The resulting DEGs between the groups were defined at cut-off criteria of |log_2_ fold-change| ≥ 1.5 and p-value < 0.05 adjusted using the Benjamini and Hochberg’s approach for controlling the false discovery rate (Liao et al., 2014). Gene set enrichment analysis (Subramanian et al., 2005) was used to assess the statistical enrichment of gene ontologies and pathways, and visualized using Clusterprofiler v4.8.1 (2012). All statistical analyses were performed using R 4.2.0 (Yu et al., 2012).

### GeoMx digital spatial profiling (DSP)

Formalin-fixed paraffin-embedded (FFPE) blocks were prepared using lymph node tissue from biopsies at baseline, week 2 post protein1, and week 3 post protein 2 immunization timepoints to analyze the spatial protein profiling using the Nanostring GeoMx Digital Spatial Profiler (DSP). Tissue sections of 5 μm thickness were cut from FFPE blocks and mounted on GeoMx-NGS BOND RX slide as per manufacturer’s recommendations. Sections were baked at 60 °C for 30 min, deparaffinized, rehydrated in CitriSolv, ethanol and washed in water. For antigen retrieval, slides were placed in a staining jar containing 1 X Citrate Buffer (pH 6) for 15 min using a preheated pressure cooker (high pressure and high temp). The tissue slides were washed with 1 X TBS-T and blocked with Buffer W for 1 hr at room temperature in a closed humidity chamber. Tissue slides were stained with a cocktail of fluorescent morphological markers SYTO13 (nuclear stain; AF532), CD20 (Clone: L26; AF488), CD3 (Clone: CD3-12; AF594), and Ki-67 (Clone: B56; AF647) at 1:10,000, 1:250, 1:100, and 1:1000 dilutions, respectively. For protein detection, a multiplex cocktail of primary antibodies was used from core panels; GeoMx immune cell profiling panel, GeoMx IO drug target module, and GeoMx immune activation status module (See Table 2). In total, 60 regions of interest (ROIs) were selected within GCs based on co-localization of CD3 with CD20 +Ki-67+GC B cells. Indexing oligos were released from each ROI by exposure to UV light as described (Merritt et al., 2020), and 10 μl of liquid from above the ROI was collected by a microcapillary tip and deposited in a 96-well plate.

Indexing oligos from each ROI were PCR amplified using GeoMx Seq Code primers. PCR products were pooled and purified twice with AMPure XP beads (Beckman Coulter, Brea, CA). Library concentration and purity were measured using a high-sensitivity DNA Bioanalyzer chip (Agilent Technologies, Santa Clara, CA). Paired-end sequencing was performed on an Illumina HiSeq 2000 instrument (Illumina, San Diego, CA). After sequencing, fastq files were run through the GeoMx NGS Pipeline where reads were trimmed, merged, and aligned to a list of indexing oligos to identify the source probe. Analysis of filtered normalized gene expression data was performed in R with Bioconductor. We profiled 32 proteins along with core protein in GeoMx Human Protein array. We first calculated the signal-to-noise ratio by dividing raw count values by the geometric mean of the negative IgG probes. The data were normalized using three negative control IgG probes with the GeomxTools package. Normalized expression values were used for downstream analyses. Dimensionality reduction analysis was performed with principal component analysis using the facomineR package (Lê et al., 2008). Differential expression analysis was conducted with the mixedModelDE function from the GeomxTools package. We tested for differences between time points and/or vaccine groups using linear mixed effect models that incorporated animal ID as a random effect term to account for non-independence of the multiple ROIs sampled per animal. Differential expression results were visualized in heatmap plots generated using the ComplexHeatmap R package (Gu et al., 2016).

### Serum IgG ELISA

Serum IgG titers against HIV-1 C.1086 Env gp140 were determined by ELISA as described previously (Verma et al., 2019). In brief, 96-well microtiter plates with high-binding capacity (Thermo Fisher, USA) were coated overnight at 4 °C with C.1086 Env gp140 protein from the NIH AIDS Reagent Program (ARP) diluted in coating buffer. Plates were washed and blocked with nonfat dry milk in PBS for two hours at room temperature and washed five times with 1 X PBS-0.05% tween-20 (PBST). Diluted standard and serum samples were added to plate wells and incubated overnight at 4 °C. Plates were washed, and HRP conjugated goat anti-monkey IgG (Nordic MUbio, Netherlands) was added to plate wells and incubated at room temperature for 1 hr. Plates were then washed and developed with the addition of TMB substrate (Thermo Fisher, USA). Absorbance was recorded at 450 nm with a reference filter at 570 nm using a Spectramax 5 plate reader (Molecular Devices, USA). Baseline sera from each animal served as negative control and optical density (OD) values twofold above baseline were considered positive and extrapolated using in-plate standards to determine anti-Env antibody concentrations.

### IgG subclass antibodies

ELISA was used to measure concentrations of gp120-specific IgG1-4 antibodies. Ten rows of a 96-well Immulon 4 microtiter plate (VWR) were coated overnight at 4 °C with 50 ng C.1086 gp120Δ7 (HIV Reagent Program) per well in PBS, pH 7.2. To generate a standard curve with a known amount of IgG1, IgG2, IgG3 or IgG4 antibody, 2 rows of the plate were coated with 50 ng per well recombinant CD40 (NHP Reagent Resource). The following day, the plate was washed with PBST and blocked with PBST containing 0.1% BSA (ELISA buffer: EB). Serum samples diluted in EB were then added to gp120 wells. Duplicate dilutions of anti-CD40 rhesus IgG1, IgG2, IgG3, or IgG4 antibody (NHP Reagent Resource) were added to the CD40 wells. Following overnight incubation at 4 °C, the plate was washed and treated for 1 hr at 37 °C with 0.5 µg/ml of the appropriate biotinylated monoclonal antibody (all from NHP Reagent Resource) diluted in EB: anti-rhesus IgG1 clone 7H11 (ena), anti-rhesus IgG2 clone 8D11 (dio), anti-rhesus IgG3 clone 6F5 (tria) or anti-rhesus IgG4 clone 7A8 (tessera). Plates were washed, treated with 1/4,000 diluted neutralite avidin peroxidase (SouthernBiotech) for 30 min at room temperature, developed with TMB substrate and quenched with a H_2_SO_4_ stop solution. After recording absorbance at 450 nm, a standard curve constructed from the anti-CD40 IgG subclass antibody was used to interpolate the concentration of anti-gp120 IgG of the same subclass in samples.

### Avidity index binding antibody multiplex assay

A binding antibody multiplex assay avidity index (BAMA-AI) method (Buchbinder et al., 2017; Seaton et al., 2021) was used to measure the strength of IgG antibody-antigen interactions in serum collected at baseline and 0-, 2-, 8-, and 12 weeks post second protein boost. Baseline sera were diluted at 1:80, and post-immunization sera were diluted at 1:80 and titrated 5-fold for 6 dilutions. Diluted serum samples were incubated with a mixture of magnetic bead sets coupled to one of five HIV-1 antigens C.1086 gp140, C.1086_gp120, C.1086_V1V2, Con C (clade C consensus) gp140, Con S (group M consensus) gp140 for 30 min. Beads were then washed and treated with dissociative sodium citrate buffer (pH 4.0, Teknova) or PBS for 15 min, prior to addition of a goat anti-monkey IgG-biotin secondary detection antibody (4 µg/mL; Rockland) for 30 min followed by phycoerythrin (PE) streptavidin (1:100 dilution; BD Pharmingen) for 30 minutes. Beads were acquired on a Bio-Plex 200 instrument (Bio-Rad), with antibody binding expressed as mean fluorescence intensity (MFI). Positive controls included HIV IgG immunoglobulin (HIVIG) and CH58_4 A IgG (HIV-1 V2-specific) monoclonal antibody titrations. Negative controls included blank (uncoupled) beads, normal human reference serum (Sigma-Aldrich), and pooled seronegative monkey plasma (BioIVT). Titer of HIV-1-specific binding antibodies was reported as AUC, calculated across the titrated PBS-treated wells. Avidity of antibody binding was reported as avidity index (a percentage), defined as MFI in the citrate treated well divided by MFI in PBS-treated well x 100. AI was calculated only if the response in the PBS-treated well was considered positive and in the linear range of the assay. Positivity criteria were as follows: (1) MFI >100 (2) MFI >antigen-specific cut-off (95th percentile of all baseline sample binding per antigen after high baseline exclusion), (3) MFI >threefold of matched baseline sample binding before and after blank bead subtraction. Positivity calls were made at the 1:80 dilution for samples tested in PBS. AI values were confirmed to be within 10% across the sample dilutions, with the lowest dilution meeting this criterion selected for reporting. Samples that did not meet the above-mentioned criteria were reported as indeterminate for AI.

### ADP assay

Serum antibodies were evaluated for ability to enhance phagocytosis of gp120 expressing fluorescent beads by THP-1 monocytes as previously described (Verma et al., 2019). Briefly, 1 µm avidin-coated fluorospheres (Invitrogen) were labeled with biotinylated anti-His tag monoclonal antibody (ThermoScientific) and used to capture His-tagged clade C gp120 Du151 protein (Immune Technologies). The gp120-expressing beads were then incubated with triplicate fivefold dilutions of heat-inactivated serum samples in V-bottom plates for 1 hr at 37 °C. THP-1 cells (2x10^4^ per well) were then added and incubated at 37 °C in 5% CO_2_. After 5 h, cells were washed with Ca^+2/^Mg^+2^-free DPBS, then treated with 0.12% trypsin-EDTA for 5 min at 37 °C. The cells were washed and resuspended in 1% paraformaldehyde. Fluorescence was measured using a FACSCanto (BD Biosciences) and FlowJo 9 software. Phagocytosis was quantified by multiplying the percentage of fluorescent cells by their median fluorescence intensity. A phagocytic score was then calculated by dividing the phagocytosis measured in each test sample by the phagocytosis observed for pooled pre-immune serum at the same dilution.

### Neutralization assay

Serum neutralizing activity was quantified using a previously described pseudovirus infectivity assay where TZM-bl cells were co-incubated with tier 1 clade C pseudovirus MW965.26, tier 2 clade C virus Ce1086_B2, tier 2 clade C Ce1176_A3, or a control MLV-pseudotyped virus (to measure non-HIV-specific activity). Neutralization titers were measured in serum samples collected at week 0 and week 2 following the second protein boost. Samples neutralizing antibody activity against SIV pseudoviruses was measured relative to MLV pseudovirus negative controls.

### Mucosal antibodies

A custom binding antibody multiplex assay (BAMA) assay with C.1086 gp140 K160N-conjugated Bio-Rad Bio-plex Pro magnetic COOH beads was used to quantify Env-specific IgG in rectal secretions and specific IgA in IgG-depleted sera and rectal secretions as previously described (Verma et al., 2019). Briefly, 50 µg gp140 was conjugated to 10x10^6^ beads as described (Iyer et al., 2015). The gp140-labeled beads (3500 per well) were shaken overnight at 1100 rpm and 4 °C with 10-fold dilutions of serum or secretion and standard Verma et al., 2019. Beads were then washed with PBST and reacted for 30 minutes at room temperature with biotinylated goat anti-human IgG (Southern Biotech) or goat whole IgG containing anti-monkey IgA (Novus) followed by phycoerythrin-labeled neutralite avidin (Southern Biotech). Fluorescence was measured with a Bio-Rad Bioplex 200. Antibody concentrations were determined using standard curves prepared with Bioplex Manager software (Bio-Rad). Concentrations of gp120-specific IgG in secretions were divided by the concentration of total IgG or IgA measured in the sample by ELISA to obtain the specific activity (ng of gp140-specific IgG or IgA antibody per µg of total IgG or IgA, respectively).

### Statistical analysis

Statistical analysis was performed using Prism v.9.5.1 (Graph Pad). Gaussian distribution was not assumed, thus comparisons across time, between groups, and correlations were analyzed using non-parametric tests. For within group comparisons, Wilcoxon matched pairs signed rank test was used. For between group comparisons, Mann-Whitney rank comparisons were used. Correlation coefficients were computed using Spearman correlation. p-values <0.05 were considered significant.

## Data Availability

RNA-seq dataset is accessible at GSE234813. The following dataset was generated: IyerSS VermaA 2023CD4 T Follicular Helper 1 Cells Promote HIV-1 Env Antibody PersistenceNCBI Gene Expression OmnibusGSE234813

## Additional files

MDAR checklist

## References

[bib1] Alving CR, Peachman KK, Rao M, Reed SG (2012). Adjuvants for human vaccines. Current Opinion in Immunology.

[bib2] Barbet G, Sander LE, Geswell M, Leonardi I, Cerutti A, Iliev I, Blander JM (2018). Sensing microbial viability through bacterial RNA augments T follicular helper cell and antibody responses. Immunity.

[bib3] Buchbinder SP, Grunenberg NA, Sanchez BJ, Seaton KE, Ferrari G, Moody MA, Frahm N, Montefiori DC, Hay CM, Goepfert PA, Baden LR, Robinson HL, Yu X, Gilbert PB, McElrath MJ, Huang Y, Tomaras GD, HIV Vaccine Trials Network (HVTN) 094 Study Group (2017). Immunogenicity of a novel Clade B HIV-1 vaccine combination: Results of phase 1 randomized placebo controlled trial of an HIV-1 GM-CSF-expressing DNA prime with a modified vaccinia Ankara vaccine boost in healthy HIV-1 uninfected adults. PLOS ONE.

[bib4] Carpenter MC, Ackerman ME (2020). Recent insights into Fc-mediated effector responses to HIV-1. Current Opinion in HIV and AIDS.

[bib5] Christensen D, Bøllehuus Hansen L, Leboux R, Jiskoot W, Christensen JP, Andersen P, Dietrich J (2019). A liposome-based adjuvant containing two delivery systems with the ability to induce mucosal immunoglobulin a following a parenteral immunization. ACS Nano.

[bib6] Cohen KW, Tian Y, Thayer C, Seese A, Amezquita R, McElrath MJ, De Rosa SC, Gottardo R (2022). Th2-biased transcriptional profile predicts HIV envelope-specific polyfunctional CD4^+^ T Cells that correlated with reduced risk of infection in RV144 trial. Journal of Immunology.

[bib7] Crotty S (2014). T follicular helper cell differentiation, function, and roles in disease. Immunity.

[bib8] Davidsen J, Rosenkrands I, Christensen D, Vangala A, Kirby D, Perrie Y, Agger EM, Andersen P (2005). Characterization of cationic liposomes based on dimethyldioctadecylammonium and synthetic cord factor from M. tuberculosis (trehalose 6,6’-dibehenate)-a novel adjuvant inducing both strong CMI and antibody responses. Biochimica et Biophysica Acta.

[bib9] Dobin A, Davis CA, Schlesinger F, Drenkow J, Zaleski C, Jha S, Batut P, Chaisson M, Gingeras TR (2013). STAR: ultrafast universal RNA-seq aligner. Bioinformatics.

[bib10] Eisinger RW, Fauci AS (2018). Ending the HIV/AIDS Pandemic1. Emerging Infectious Diseases.

[bib11] Fabregat A, Sidiropoulos K, Viteri G, Forner O, Marin-Garcia P, Arnau V, D’Eustachio P, Stein L, Hermjakob H (2017). Reactome pathway analysis: a high-performance in-memory approach. BMC Bioinformatics.

[bib12] Fauci AS (2017). An HIV vaccine is essential for ending the HIV/AIDS pandemic. JAMA.

[bib13] Felber BK, Lu Z, Hu X, Valentin A, Rosati M, Remmel CAL, Weiner JA, Carpenter MC, Faircloth K, Stanfield-Oakley S, Williams WB, Shen X, Tomaras GD, LaBranche CC, Montefiori D, Trinh HV, Rao M, Alam MS, Vandergrift NA, Saunders KO, Wang Y, Rountree W, Das J, Alter G, Reed SG, Aye PP, Schiro F, Pahar B, Dufour JP, Veazey RS, Marx PA, Venzon DJ, Shaw GM, Ferrari G, Ackerman ME, Haynes BF, Pavlakis GN (2020). Co-immunization of DNA and protein in the same anatomical sites induces superior protective immune responses against SHIV challenge. Cell Reports.

[bib14] Fischinger S, Boudreau CM, Butler AL, Streeck H, Alter G (2019). Sex differences in vaccine-induced humoral immunity. Seminars in Immunopathology.

[bib15] Gao X, Luo K, Wang D, Wei Y, Yao Y, Deng J, Yang Y, Zeng Q, Dong X, Xiong L, Gong D, Lin L, Pohl K, Liu S, Liu Y, Liu L, Nguyen THO, Allen LF, Kedzierska K, Jin Y, Du MR, Chen W, Lu L, Shen N, Liu Z, Cockburn IA, Luo W, Yu D (2023). T follicular helper 17 (Tfh17) cells are superior for immunological memory maintenance. eLife.

[bib16] Gu Z, Eils R, Schlesner M (2016). Complex heatmaps reveal patterns and correlations in multidimensional genomic data. Bioinformatics.

[bib17] Hayashi N, Yoshimoto T, Izuhara K, Matsui K, Tanaka T, Nakanishi K (2007). T helper 1 cells stimulated with ovalbumin and IL-18 induce airway hyperresponsiveness and lung fibrosis by IFN-gamma and IL-13 production. PNAS.

[bib18] Henriksen-Lacey M, Christensen D, Bramwell VW, Lindenstrøm T, Agger EM, Andersen P, Perrie Y (2011). Comparison of the depot effect and immunogenicity of liposomes based on dimethyldioctadecylammonium (DDA), 3β-[N-(N’,N’-Dimethylaminoethane)carbomyl] cholesterol (DC-Chol), and 1,2-Dioleoyl-3-trimethylammonium propane (DOTAP): prolonged liposome retention mediates stronger Th1 responses. Molecular Pharmaceutics.

[bib19] Hill DL, Pierson W, Bolland DJ, Mkindi C, Carr EJ, Wang J, Houard S, Wingett SW, Audran R, Wallin EF, Jongo SA, Kamaka K, Zand M, Spertini F, Daubenberger C, Corcoran AE, Linterman MA (2019). The adjuvant GLA-SE promotes human Tfh cell expansion and emergence of public TCRβ clonotypes. The Journal of Experimental Medicine.

[bib20] Hirota K, Turner JE, Villa M, Duarte JH, Demengeot J, Steinmetz OM, Stockinger B (2013). Plasticity of Th17 cells in Peyer’s patches is responsible for the induction of T cell-dependent IgA responses. Nature Immunology.

[bib21] Iyer SS, Gangadhara S, Victor B, Gomez R, Basu R, Hong JJ, Labranche C, Montefiori DC, Villinger F, Moss B, Amara RR (2015). Codelivery of envelope protein in alum with mva vaccine induces CXCR3-Biased CXCR5+ and CXCR5- CD4 T cell responses in rhesus macaques. Journal of Immunology.

[bib22] Kumar P, Chen K, Kolls JK (2013). Th17 cell based vaccines in mucosal immunity. Current Opinion in Immunology.

[bib23] Lê S, Josse J, Husson F (2008). FactoMineR: an R package for multivariate analysis. Journal of Statistical Software.

[bib24] Liao Y, Smyth GK, Shi W (2014). featureCounts: an efficient general purpose program for assigning sequence reads to genomic features. Bioinformatics.

[bib25] Lin L, Finak G, Ushey K, Seshadri C, Hawn TR, Frahm N, Scriba TJ, Mahomed H, Hanekom W, Bart PA, Pantaleo G, Tomaras GD, Rerks-Ngarm S, Kaewkungwal J, Nitayaphan S, Pitisuttithum P, Michael NL, Kim JH, Robb ML, O’Connell RJ, Karasavvas N, Gilbert P, C De Rosa S, McElrath MJ, Gottardo R (2015). COMPASS identifies T-cell subsets correlated with clinical outcomes. Nature Biotechnology.

[bib26] Love MI, Huber W, Anders S (2014). Moderated estimation of fold change and dispersion for RNA-seq data with DESeq2. Genome Biology.

[bib27] Martínez-López M, Iborra S, Conde-Garrosa R, Mastrangelo A, Danne C, Mann ER, Reid DM, Gaboriau-Routhiau V, Chaparro M, Lorenzo MP, Minnerup L, Saz-Leal P, Slack E, Kemp B, Gisbert JP, Dzionek A, Robinson MJ, Rupérez FJ, Cerf-Bensussan N, Brown GD, Bernardo D, LeibundGut-Landmann S, Sancho D (2019). Microbiota sensing by mincle-Syk axis in dendritic cells regulates interleukin-17 and -22 production and promotes intestinal barrier integrity. Immunity.

[bib28] McCausland MM, Yusuf I, Tran H, Ono N, Yanagi Y, Crotty S (2007). SAP regulation of follicular helper CD4 T cell development and humoral immunity is independent of SLAM and Fyn kinase. Journal of Immunology.

[bib29] Merritt CR, Ong GT, Church SE, Barker K, Danaher P, Geiss G, Hoang M, Jung J, Liang Y, McKay-Fleisch J, Nguyen K, Norgaard Z, Sorg K, Sprague I, Warren C, Warren S, Webster PJ, Zhou Z, Zollinger DR, Dunaway DL, Mills GB, Beechem JM (2020). Multiplex digital spatial profiling of proteins and RNA in fixed tissue. Nature Biotechnology.

[bib30] Mitsdoerffer M, Lee Y, Jäger A, Kim HJ, Korn T, Kolls JK, Cantor H, Bettelli E, Kuchroo VK (2010). Proinflammatory T helper type 17 cells are effective B-cell helpers. PNAS.

[bib31] Morita R, Schmitt N, Bentebibel SE, Ranganathan R, Bourdery L, Zurawski G, Foucat E, Dullaers M, Oh S, Sabzghabaei N, Lavecchio EM, Punaro M, Pascual V, Banchereau J, Ueno H (2011). Human blood CXCR5(+)CD4(+) T cells are counterparts of T follicular cells and contain specific subsets that differentially support antibody secretion. Immunity.

[bib32] Moyron-Quiroz JE, Rangel-Moreno J, Kusser K, Hartson L, Sprague F, Goodrich S, Woodland DL, Lund FE, Randall TD (2004). Role of inducible bronchus associated lymphoid tissue (iBALT) in respiratory immunity. Nature Medicine.

[bib33] Olatunde AC, Hale JS, Lamb TJ (2021). Cytokine-skewed Tfh cells: functional consequences for B cell help. Trends in Immunology.

[bib34] Ovsyannikova IG, Dhiman N, Jacobson RM, Vierkant RA, Poland GA (2003a). Frequency of measles virus-specific CD4+ and CD8+ T cells in subjects seronegative or highly seropositive for measles vaccine. Clinical and Diagnostic Laboratory Immunology.

[bib35] Ovsyannikova IG, Reid KC, Jacobson RM, Oberg AL, Klee GG, Poland GA (2003b). Cytokine production patterns and antibody response to measles vaccine. Vaccine.

[bib36] Pedersen GK, Andersen P, Christensen D (2018). Immunocorrelates of CAF family adjuvants. Seminars in Immunology.

[bib37] Pedersen GK, Wørzner K, Andersen P, Christensen D (2020). Vaccine adjuvants differentially affect kinetics of antibody and germinal center responses. Frontiers in Immunology.

[bib38] Rao M, Onkar S, Peachman KK, White Y, Trinh HV, Jobe O, Zhou Y, Dawson P, Eller MA, Matyas GR, Alving CR (2018). liposome-encapsulated human immunodeficiency virus-1 gp120 induces potent V1V2-specific antibodies in humans. The Journal of Infectious Diseases.

[bib39] Seaton KE, Spreng RL, Abraha M, Reichartz M, Rojas M, Feely F, Huntwork RHC, Dutta S, Mudrak SV, Alam SM, Gregory S, Jongert E, Coccia M, Ulloa-Montoya F, Wille-Reece U, Tomaras GD, Dennison SM (2021). Subclass and avidity of circumsporozoite protein specific antibodies associate with protection status against malaria infection. NPJ Vaccines.

[bib40] Slifka MK, Antia R, Whitmire JK, Ahmed R (1998). Humoral immunity due to long-lived plasma cells. Immunity.

[bib41] Subramanian A, Tamayo P, Mootha VK, Mukherjee S, Ebert BL, Gillette MA, Paulovich A, Pomeroy SL, Golub TR, Lander ES, Mesirov JP (2005). Gene set enrichment analysis: a knowledge-based approach for interpreting genome-wide expression profiles. PNAS.

[bib42] Verma A, Schmidt BA, Elizaldi SR, Nguyen NK, Walter KA, Beck Z, Trinh HV, Dinsarapu AR, Lakshmanappa YS, Rane NN, Matyas GR, Rao M, Shen X, Tomaras GD, LaBranche CC, Reimann KA, Foehl DH, Gach JS, Forthal DN, Kozlowski PA, Amara RR, Iyer SS (2019). Impact of Th1 CD4 TFH skewing on antibody responses to an HIV-1 vaccine in rhesus macaques. Journal of Virology.

[bib43] Ward BJ, Griffin DE (1993). Changes in cytokine production after measles virus vaccination: predominant production of IL-4 suggests induction of a Th2 response. Clinical Immunology and Immunopathology.

[bib44] Werninghaus K, Babiak A, Gross O, Hölscher C, Dietrich H, Agger EM, Mages J, Mocsai A, Schoenen H, Finger K, Nimmerjahn F, Brown GD, Kirschning C, Heit A, Andersen P, Wagner H, Ruland J, Lang R (2009). Adjuvanticity of a synthetic cord factor analogue for subunit Mycobacterium tuberculosis vaccination requires FcRgamma-Syk-Card9-dependent innate immune activation. The Journal of Experimental Medicine.

[bib45] Wørzner K, Hvannastein J, Schmidt ST, Foged C, Rosenkrands I, Pedersen GK, Christensen D (2021). Adsorption of protein antigen to the cationic liposome adjuvant CAF01 is required for induction of Th1 and Th17 responses but not for antibody induction. European Journal of Pharmaceutics and Biopharmaceutics.

[bib46] Yu G, Wang LG, Han Y, He QY (2012). ClusterProfiler: an R package for comparing biological themes among gene clusters. Omics.

